# The effect of implementation strength of basic emergency obstetric and newborn care (BEmONC) on facility deliveries and the met need for BEmONC at the primary health care level in Ethiopia

**DOI:** 10.1186/s12884-018-1751-z

**Published:** 2018-05-02

**Authors:** Gizachew Tadele Tiruneh, Ali Mehryar Karim, Bilal Iqbal Avan, Nebreed Fesseha Zemichael, Tewabech Gebrekiristos Wereta, Deepthi Wickremasinghe, Zinar Nebi Keweti, Zewditu Kebede, Wuleta Aklilu Betemariam

**Affiliations:** 1 The Last Ten Kilometers (L10K) Project, JSI Research & Training Institute, Inc., Addis Ababa, Ethiopia; 2 The Last Ten Kilometers (L10K) Project, JSI Research & Training Institute, Inc., Washington DC, USA; 3 IDEAS project, London School of Hygiene & Tropical Medicine, London, UK; 4 United States Agency for International Development (USAID), Addis Ababa, Ethiopia

**Keywords:** Basic emergency obstetric and newborn care (BEmONC), Emergency obstetric care (EmONC), Ethiopia, Implementation strength, Life-saving interventions, Low- and middle-income countries (LMIC), Maternal and newborn health, Primary health care, Rural health centers

## Abstract

**Background:**

Basic emergency obstetric and newborn care (BEmONC) is a primary health care level initiative promoted in low- and middle-income countries to reduce maternal and newborn mortality. Tailored support, including BEmONC training to providers, mentoring and monitoring through supportive supervision, provision of equipment and supplies, strengthening referral linkages, and improving infection-prevention practice, was provided in a package of interventions to 134 health centers, covering 91 rural districts of Ethiopia to ensure timely BEmONC care. In recent years, there has been a growing interest in measuring program implementation strength to evaluate public health gains. To assess the effectiveness of the BEmONC initiative, this study measures its implementation strength and examines the effect of its variability across intervention health centers on the rate of facility deliveries and the met need for BEmONC.

**Methods:**

Before and after data from 134 intervention health centers were collected in April 2013 and July 2015. A BEmONC implementation strength index was constructed from seven input and five process indicators measured through observation, record review, and provider interview; while facility delivery rate and the met need for expected obstetric complications were measured from service statistics and patient records. We estimated the dose–response relationships between outcome and explanatory variables of interest using regression methods.

**Results:**

The BEmONC implementation strength index score, which ranged between zero and 10, increased statistically significantly from 4.3 at baseline to 6.7 at follow-up (*p* < .05). Correspondingly, the health center delivery rate significantly increased from 24% to 56% (*p* < .05). There was a dose–response relationship between the explanatory and outcome variables. For every unit increase in BEmONC implementation strength score there was a corresponding average of 4.5 percentage points (95% confidence interval: 2.1–6.9) increase in facility-based deliveries; while a higher score for BEmONC implementation strength of a health facility at follow-up was associated with a higher met need.

**Conclusion:**

The BEmONC initiative was effective in improving institutional deliveries and may have also improved the met need for BEmONC services. The BEmONC implementation strength index can be potentially used to monitor the implementation of BEmONC interventions.

**Electronic supplementary material:**

The online version of this article (10.1186/s12884-018-1751-z) contains supplementary material, which is available to authorized users.

## Background

Globally, about 45% of maternal deaths and 36% of neonatal deaths occur during the first 24 h after birth [1, 2]. Maternal and newborn deaths related to the perinatal period are largely preventable, and most life-threatening conditions are treatable if skilled health care is provided during the intrapartum and early postnatal periods [3–5] at the primary health care level [6–8]. However, in low- and middle-income countries (LMIC), where maternal and neonatal mortality is high, the availability, accessibility, and use of proven life-saving interventions for the treatment of obstetric complications are low [9, 10].

Ethiopia made progress in improving maternal and child health during the Millennium Development Goal era from 1990 to 2015. The maternal mortality rate (MMR) dropped by 72% from 1250 to 353 per 100,000 live births, while child mortality decreased by two-thirds from 205 to 59 per 1000 live births [10, 11]. The reduction in the neonatal mortality rate (NMR) has been slow during that period and is currently 28 deaths per 1000 live births, accounting for 47% of all under-5 years mortality [11]. Despite this decline, Ethiopia’s MMR is still regarded as among the highest in the world [10]. Moreover, the coverage of skilled attendance at birth and the met need for obstetric and newborn care are low [12, 13]. Committed to contribute toward achieving the maternal and newborn survival-related Sustainable Development Goals (i.e., to reduce global MMR to less than 70 per 100,000 live births and NMR to at least as low as 12 per 1000 live births by 2030 [14, 15], the Health Sector Transformation Plan (HSTP) of Ethiopia targets to reduce MMR to 199 per 100,000 live births and NMR to 10 per 1000 live births by 2020 [16].

One of the key strategies promoted to reduce both maternal and newborn mortality in LMIC is the timely access to comprehensive emergency obstetric and newborn care (CEmONC) [5, 17], with the provision of at least the basic emergency obstetric and newborn care (BEmONC) (see Box 1 for definition) at the primary health care level of a country’s health system [17]. To track progress in monitoring this strategy, the availability of BEmONC services, the proportion of all live births taking place in facilities, and the met need for EmONC are among the key indicators that have been developed for the primary care level [17] (Table 1).

**Table 1:** Definition of BEmONC

Emergency obstetric and newborn care (EmONC) is defined as a set of life-saving interventions, that treat the major obstetric and newborn causes of morbidity and mortality. To assess the level of care, these functions are classified as basic EmONC (BEmONC) or comprehensive EmONC (CEmONC) levels of care. BEmONC services comprise: 1) administration of parenteral antibiotics to prevent puerperal infection or treat abortion complications; 2) administration of parenteral anticonvulsants for treatment of eclampsia and preeclampsia; 3) administration of parenteral uterotonic drugs for postpartum hemorrhage; 4) manual removal of the placenta; 5) assisted vaginal delivery (vacuum extractions); 6) removal of retained products of conception; and 7) neonatal resuscitation. If a facility has provided all seven key BEmONC functions in the last 3 months, it is called a BEmONC facility. CEmONC services comprise caesarean sections and blood transfusions, in addition to BEmONC functions. *Source: WHO, 2009* [17]	

A systematic review of studies using quasi-experimental, observational, and ecological designs demonstrates evidence of the effectiveness of EmONC interventions in reducing maternal mortality in LMIC [3]. A recent intervention study in Ethiopia suggests that upgrading facilities to provide life-saving interventions decreased the MMR and improved facility delivery rate [18]. Similar studies elsewhere demonstrate that implementation of EmONC has significantly improved the facility delivery and the met need for EmONC [19].

Despite the availability of evidence of the efficacy of life-saving interventions to avert high rates of maternal and newborn deaths [5, 20], there is little understanding of how to deliver those interventions effectively [21–23]. Accordingly, intervention strategies need to be properly evaluated to identify successful, evidence-based, low-cost interventions, or implementation approaches that can be scaled-up, to promote their sustainable integration into health systems, and to ensure accountability to donors for public health gains [22–24]. Measuring the strength of program implementation is one of the evaluation approaches that helps to understand which packages of interventions are delivered effectively (and which are not) and why some programs are effective and attribute outcomes to program implementation characteristics [23, 25, 26]. However, in LMIC, there is a gap between knowledge of proven interventions and gains through implementation variations [22].

Strengthening the health care system to provide BEmONC is a key priority for providing life-saving services, especially in rural areas [12, 18, 27]. The Government of Ethiopia took an initiative to improve access to BEmONC services by upgrading the capability of health centers at the country’s primary health care level. Measuring the implementation strength of the BEmONC package of interventions (i.e., the degree of functionality of the different aspects of BEmONC) at the health centers in Ethiopia is essential to understanding which aspects of BEmONC care improved (and which did not). Moreover, it also helped identify whether the improvement in BEmONC was associated with an increase in utilization of services for critical maternal and newborn care practices. As such, the implementation variability of BEmONC initiatives can be studied with the concept of implementation strength to explore the association between the implementation strength and utilization of facility delivery. It is hypothesized that higher implementation strength of the BEmONC score of primary level facilities is associated with the utilization of facility deliveries and the met need for BEmONC.

Most previous studies have focused on changes in the uptake of EmONC, the variability of program inputs, and effectiveness of special interventions like the implementation of transport systems, emergency loans, community financing, communications, or various combinations of these elements [18, 19, 28–30] on the uptake of facility delivery and the met need for EmONC, without distinguishing the level of care. Moreover, there is little programmatic evidence of how implementation variability affects the uptake of maternal and newborn health services at the primary health care level. Thus, this study examines the effectiveness of the BEmONC initiative by analyzing the effect of facility inputs and the process of service delivery as implementation strength index score on the rate of facility deliveries and the met need for BEmONC in rural health centers of Ethiopia.

### Study settings

The Ethiopian primary health care system employs decentralized governance structures where regions are divided into zones, which are internally divided into districts. To respond to its population needs, the Government of Ethiopia has restructured its health service into a three-tier system of care: primary, secondary, and tertiary [16]. Primary level health care, the major platform for health service delivery at the grassroots level, in a rural district in Ethiopia consists of one primary hospital with four or five primary health care units (PHCUs). Each PHCU comprises a health center with five satellite health posts serving about 25,000 people in total. Health centers are staffed with health officers, nurses, midwives, and laboratory technicians to provide primarily preventive, curative, inpatient and ambulatory services, treatment of common psychiatric disorders, and dental services.

To reduce the high rate of maternal mortality in Ethiopia, the Government expanded health centers to achieve a target of one health center providing all BEmONC functions for every 25,000 population during the implementation of Health Sector Development Plan IV from 2010 to 2015. Moreover, the country sought to improve access to, and utilization of, BEmONC services by mobilizing communities to encourage pregnant mothers to give birth in health centers, expanding health centers, staffing health centers with midwives to ensure BEmONC services are always available, and providing ambulances to districts to mitigate transportation barriers [31].

However, national evidence shows that most health centers were weak in providing life-saving BEmONC interventions, with only a few facilities providing all BEmONC functions. In most health centers, drugs, equipment, and supplies were missing, the centers were poorly staffed, or staff lacked the skills to apply most BEmONC functions. Moreover, the proportion of facility deliveries and the met need for BEmONC were low [12, 13]. Strengthening health centers to provide all BEmONC functions remains a challenge. This is particularly so in rural health centers [32]. As such, the Federal Ministry of Health has set targets for facility delivery coverage at 90% and to enable all health centers to provide all BEmONC functions by 2020 [16].

## Methods

### The BEmONC initiative

To upgrade the capability of health centers to provide timely BEmONC, tailored support was provided to 134 health centers, covering 91 rural districts of Ethiopia during the first phase of Ethiopia’s BEmONC-strengthening initiative, which was initiated in April 2013. This included BEmONC training to providers, mentoring and monitoring through post-training follow-up, provision of equipment and supplies, strengthening referral linkages, and improving infection-prevention practice.

### Study design

A before and after evaluation for the BEmONC intervention included a cross-sectional survey conducted in April 2013 and July 2015, in 134 rural health centers in 91 districts of four regions: Amhara, Oromia, the Southern National, Nationalities, and Peoples’ Region, and Tigray. The effectiveness of the BEmONC initiative was determined by the dose–response relationships between BEmONC implementation strength (BIS) of the health centers and the outcomes of interest.

### Conceptual framework

**Fig. 1:**
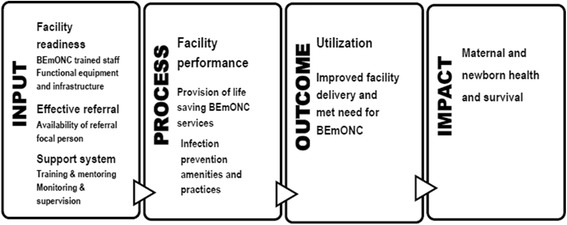
Conceptual framework: BIS components to improve critical care service utilization at primary facilities

A conceptual framework for measuring BIS was developed through a review of peer-reviewed and gray literature. Organizational structure (i.e., the resources of the health care system) and processes of service delivery are identified as components for measuring implementation strength [17, 25, 33, 34]. The structural components, well-documented in the literature, to provide quality BEmONC are a functioning facility with a skilled health service provider available around the clock and functional and adequate supplies, equipment, drugs, and infrastructure. An effective referral system is also critical to ensure a woman reaches the facility in a timely manner. The core elements of an effective referral system include formalized communication and transport, sufficiently resourced referral centers, active collaboration across all referral levels, protocols for the referrer and receiver, and accountability for providers’ performances, coupled with supportive supervision [34]. With regard to the process components, facilities must provide life-saving interventions in a timely manner to respond to women’s needs [8, 17, 35, 36]. In addition, the project implementation pathway was analyzed by reviewing project documents and the Project Management Plan. The critical components necessary for measuring BIS in rural health centers to improve the use of critical maternal and newborn care services are 1) facilities’ emergency readiness in terms of availability of services, human resources, equipment, and drugs; 2) effective referral; 3) the support system; and 4) adherence to basic life-saving interventions and quality of care (Fig. 1).

### Data

All 134 first-phase intervention health centers were visited during baseline and follow-up surveys. Data were collected through interviews, observation, and a review of patient records and service statistics. Data collectors conducted interviews with the heads of the health centers and health care providers working in the maternity units to gather information on the availability of equipment and supplies, human resources, the performance of BEmONC signal functions, and other maternal and newborn health services. Observations were made to assess the infrastructure and supplies available. Documents were reviewed to gather data on service statistics and patient records, to assess utilization of services for critical maternal, and newborn conditions. The survey questionnaire and the dataset we used for this study are presented as additional files (see Additional files 1 and 2).

Baseline data were collected using paper-based questionnaires and data were entered using Epi Info 7. The quality assurance of data entry was ensured through appropriate skip patterns and allowing only logical values data entry. The data entry was done twice and both entries were validated with each other to eliminate data entry errors. In the follow-up survey, data were captured using the Android mobile application SurveyCTO [37].

### Measurements

The independent variable of interest was BIS, which was measured during the baseline and follow-up surveys as an index using items measuring programmatic input and process indicators listed in Table 2 below. Three of the BEmONC signal functions, namely administration of parenteral uterotonics, administration of parenteral antibiotics, and administration of parenteral MgSO_4_ were dropped from the calculation of the BIS score because the availability of the drugs necessary for the provision of these functions was already included under indicator number 4 (Table 2).

**Table 2:** Indicators used to measure BIS index score and their operational definitions

SN	Indicators	Definition	Data source
I	Input indicators		
1	Number of BEmONC-trained personnel available	Number of BEmONC-trained providers (health officers, nurses, or midwives) working in the facility at the time of the survey	Interviews with facility heads
2	Number of laboratory tests available	Number of the following laboratory tests: hemoglobin/hematocrit, blood group, urine analysis, venereal disease research laboratory (VDRL), and HIV test for PMTCT available in the facility at the time of the data collection	Interviews with facility staff
3	Number of items of equipment available	Number of functional equipment, including oxygen concentrator, sphygmomanometer, vacuum extractor, suction machine, radiant heater, and Ambu-bag mask available in the facility for provision of BEmONC care at the time of the survey	Interviews with facility staff and observation
4	Number of drugs available	Number of the following drugs: intravenous (IV) uterotonics, IV fluids, Nifedipine, Hydralazine, IV antibiotics, IV MgSO_4_ and calcium gluconate available in the facility for provision of BEmONC services at the time of the survey	Interviews with facility staff and observation
5	Availability of ambulance services	Availability of ambulance service in the facility 24 h a day	Interviews with facility heads
6	Availability of maternity waiting homes	Availability of maternity waiting area/homes in or around the facility	Interviews with facility head and observation
7	Availability of a focal person for referral	Availability of a designated referral focal person to coordinate in- and out-referrals 24 h a day in the facility	Interviews with facility heads
II	Process indicators		
8	Infection-prevention amenities and practices	The following infection-prevention amenities and practices were observed at the time of the survey: clean facility compound, cleaning done after birth, availability of disinfectant solutions, disinfectant solution prepared and used correctly, availability of a container for sharps’ disposal, providers practice hand washing, quality mechanism in place for sterilization, staff use personal protective barriers, availability of a light source for vaginal procedure, enough physical space, good illumination and ventilation, and easily washable delivery floor	Interviews with facility staff and observation
9–12	Provision of BEmONC signal functions	Provision of the following life-saving BEmONC services in the past 3 months for the treatment of obstetric complications; 9) removal of retained products of conception; 10) manual removal of placenta; 11) assisted vaginal birth; and 12) neonatal resuscitation	Interviews with facility staff

As the variables are not on the same scale, they were standardized by dividing the observed sum of the individual variable scores values by their standard deviation to make comparisons of variables and then aggregated to obtain the BIS score. Thus, all the variables were given equal weights. The BIS index was recalibrated to range between zero and 10, with a higher score indicating better BIS. Cronbach’s alphas were calculated to assess the internal reliability of the 12 items in measuring the underlying construct of BIS. The possible values of alpha range between zero and one, and values exceeding .70 are regarded as acceptable [38, 39]. The Cronbach’s alpha for the 12 items was .71.

The programmatic outcome variables of interest were the facility delivery rate and the met need for BEmONC. The facility delivery rate was defined as the proportion of deliveries that took place in health facilities out of the total expected number of births in the catchment area. The expected births were calculated based on information from the catchment projected population—the population estimate of a health center based on the regional population growth rate estimate [40]—and the crude birth rate for the catchment area. This was calculated as the number of deliveries registered in health facilities in the last 12 months divided by the expected number of live births in that period and multiplied by 100. The facility delivery rate was calculated during the baseline and follow-up surveys. The met need for BEmONC was defined as the proportion of women with direct obstetric complications (including abortion complications, postpartum hemorrhage, obstructed or prolonged labor, and puerperal sepsis) treated at health centers in the last 12 months. It was calculated as the number of women with obstetric complications treated at health centers in the last 12 months divided by the estimated number of women who would have obstetric complications (i.e., 15% of expected births) [17] and multiplied by 100. The met need was measured only during the follow-up survey, as the information about obstetric complications was not collected during the baseline survey.

### Statistical analysis

Data were analyzed for both descriptive and inferential statistics using Stata version 14.2 [41]. Descriptive statistics were used to analyze the inputs and process of service delivery. A paired *t*-test was used to test the statistical significance of the changes in the indicators of interest between the baseline and follow-up surveys. An internal comparison group analysis was also done to assess dose–response relationships between BIS and the outcomes of interest, such as whether health centers with higher improvements in BIS were associated with increased facility delivery and the met need for BEmONC. Fixed-effects ordinary least-squares regression was used to assess the dose–response relationship between the changes in BIS found in the follow-up survey, compared with the baseline survey, and the changes in the health center delivery rate during the same period. Because information for the met need was available only at one point in time, during the follow-up survey, the cross-sectional dose–response relationship between BIS and met need was assessed using ordinary least-squares regression. A scatter plot was used to visualize the associations.

## Results

The findings are presented in four sections: 1) change in BEmONC services over time, 2) changes in BIS and service utilization over time, 3) relationship between changes in BIS over time and changes in facility birth, and 4) relationship between changes in BIS over time and the met need for BEmONC at the primary health care level.

### Change in BEmONC services over time at the primary health care level in Ethiopia

Facility input indicators, including the availability of BEmONC-trained providers, availability of essential drugs and equipment, laboratory tests, and facility infrastructure, increased statistically significantly (*p* < .05) over the study period (Table 3). The mean number of BEmONC-trained providers increased significantly from 1.4 at baseline to 2.6 during the follow-up survey (*p* < .05). Overall, the average number of laboratory tests available showed significant improvement over the survey periods with urine analyses and VDRL syphilis tests having improved significantly over time; however, hematological tests (hemoglobin and blood group) and HIV tests for PMTCT did not show statistically significant changes over time. Availability of individual drugs in the health centers showed significant positive changes for most drug categories except IV fluids and Nifedipine (*p* < .05). The largest change was observed for MgSO_4_, which increased by 65 percentage points; *p* < .01) followed by Hydralazine (53 percentage points; *p* < .01), and IV antibiotics (21 percentage points; *p* < .01). Likewise, the availability of medical equipment, including vacuum extractor sets, radiant heaters, and oxygen concentrators, showed significant change. As the values for the availability of IV fluids and sphygmomanometers were already high in the baseline survey, it would be difficult to see much improvement during the follow-up period; however, the availability of an ambulance service and a focal person to coordinate referrals showed significant improvements over the study period.

**Table 3:** Input indicators measuring BIS at baseline and follow-up in 134 health centers

Indicator	Baseline	Follow-up	Change between baseline and follow-up		
			Change	(95% CI)	*p*-value
Average number of BEmONC-trained providers	1.4	2.6	1.2	(0.9, 1.5)	<.001
Availability of laboratory tests (%)					
Hemoglobin/Hematocrit	71.6	74.6	3.0	(−0.1, 0.1)	.466
Blood group	91.0	93.3	2.3	(−0.0, 0.1)	.439
Urine analysis	79.9	94.0	14.1	(0.1, 0.2)	<.001
VDRL for syphilis test	53.7	71.6	17.9	(0.1, 0.3)	<.001
HIV test for PMTCT	99.2	95.5	−3.7	(−0.1, 0.0)	.056
*Average number of laboratory tests available*	3.9	4.3	0.4	(0.1, 0.6)	.001
Availability of equipment (%)					
Sphygmomanometer	96.3	98.5	2.2	(−0.0, 0.1)	.256
Oxygen concentrator	15.7	37.3	21.6	(0.1, 0.3)	<.001
Vacuum extractor (sets)	37.3	74.6	37.3	(0.3, 0.5)	<.001
Suction machine	44.8	58.2	13.4	(0.0, 0.3)	.025
Radiant heater	33.6	57.5	23.9	(0.1, 0.3)	<.001
Ambu-bag & masks	88.8	97.8	9.0	(0.0, 0.2)	.002
*Average number of items of equipment available*	3.2	4.2	1.0	(0.8, 1.4)	<.001
Availability of drugs (%)					
IV Uterotonics	31.3	98.5	67.2	(0.6, 0.8)	<.001
IV fluids	98.5	98.5	0.0	(−0.0, 0.0)	1.000
Nifedipine	62.7	56.7	−6.0	(−0.2, 0.1)	.267
Hydralazine	35.1	88.1	53.0	(0.4, 0.6)	<.001
IV antibiotics	78.4	99.3	20.9	(0.1, 0.3)	<.001
IV MgSO4	10.4	75.4	65.0	(0.6, 0.7)	<.001
Calcium gluconate	4.5	15.7	11.2	(0.0, 0.2)	.002
*Average number of drugs available*	3.2	5.3	2.1	(1.9, 2.4)	<.001
Availability of maternity waiting area/ homes (%)	31.3	73.9	42.6	(0.4, 0.6)	<.001
Availability ambulance service (%)	81.3	91.0	9.7	(0.4, 0.9)	<.001
Availability of referral focal person (%)	27.5	39.6	12.1	(2.4, 3.0)	<.001

The average number of infection-prevention amenities showed significant improvement over time (Table 4). The hand-washing practice of providers (42 percentage points increase, *p* < .01) and consistent use of personal protective barriers by staff (26 percentage points increase, *p* < .01) were among the larger changes seen. Illumination and ventilation of the maternity units significantly decreased over time (*p* < .05). This might be due to the difference in survey periods, as the follow-up period was in the rainy season; there might be frequent power failures as well as facilities might blind ventilation outlets to warm maternity units.

**Table 4:** Process indicators measuring BIS at baseline and follow-up in 134 health centers

Indicator	Baseline	Follow-up	Change between baseline and follow-up		
			Change	(95% CI)	*p*-value
Practice to infection-prevention measures (%)					
Facility compound clean	80.5	85.8	5.3	(−0.0, 0.2)	.262
Cleaning done after attending birth	85.1	93.3	8.2	(0.0, 0.2)	.032
Availability of disinfectant solutions	92.5	100	7.5	(0.0, 0.1)	.001
Disinfectant solution prepared & used per standard	88.0	96.3	8.3	(0.0, 0.2)	.014
Availability of container for disposing of sharps	95.5	97.8	2.3	(−0.0, 0.1)	.256
Providers’ practice hand washing	37.0	79.1	42.1	(0.3, 0.5)	<.001
Quality mechanism in place for sterilization	63.9	79.9	16	(0.1, 0.3)	.002
Staff use personal protective barrier	58.2	84.3	26.1	(0.2, 0.4)	<.001
Availability of light source for vaginal procedure	53.0	62.7	9.7	(−0.0, 0.2)	.144
Enough physical space	66.9	64.9	−2.0	(−0.1, 0.1)	.782
Good illumination and ventilation	89.5	79.9	−9.6	(−0.2, − 0.0)	.030
Easily washable delivery floor	70.7	77.6	6.9	(−0.0, 0.2)	.207
*Average number of infection-prevention amenities*	8.7	10.0	1.3	(0.9, 1.8)	<.001
Removal of retained products of conception	57.5	71.6	14.1	(0.0, 0.2)	<.05
Manual removal of placenta	72.4	75.4	3.0	(−0.1, 0.1)	.565
Assisted vaginal birth	28.4	59.0	30.6	(0.2, 0.4)	<.001
Neonatal resuscitation	77.6	78.4	0.8	(−0.1, 0.1)	.873

Statistically, significant improvements were seen in health center performance for removing retained products of conception and assisted vaginal birth. However, there were no statistically significant changes in the performance of manual placenta removal and neonatal resuscitation.

### Changes in the BIS and service utilization over time at the primary health care level in Ethiopia

**Fig. 2:**
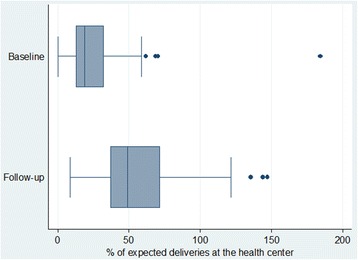
Distribution of expected deliveries at the health centers by study period

The BIS score ranged between zero and 10; which increased (*p* < .05) from 4.3 (ranging from 0 to 8 at baseline) to 6.7 (ranging from 2 to 10 at follow-up) (Table 5). Correspondingly, the health center delivery rate increased (*p* < .001) from 24% to 56%. The distribution of health center delivery rates is presented in Fig. 2 below. As shown in the box plot, 50% of health centers had a delivery rate between 12.9 and 32.3 at baseline and between 37.2 and 71.8 at follow-up; while one (0.7%) health center at baseline and 11 (8%) health centers at follow-up had delivery rates above 100%, which might be due to mothers from outside of their catchment area coming to use the service at these health centers.

**Table 5:** Changes in mean service utilization rate and BIS score at baseline and follow-up^a^

Indicator	Baseline mean (SD)	Follow-up mean (SD)	Change between baseline and follow-up		
			Mean (SD)	(95% CI)	*p*-value
BIS score	4.3 (1.8)	6.7 (1.8)	2.5 (2.1)	(2.1, 2.8)	<.001
Health center delivery	23.6 (19.6)	56.0 (27.6)	32.4 (30.8)	(27.1, 37.5)	<.001
Met need for BEmONC	–	16.0 (16.4)	–	–	–

^a^in 134 health centers

The met need for BEmONC was found to be 16% during the follow-up survey. The most frequent obstetric complication observed in this study was abortion complications, which accounted for about half (8.2%) of the complications managed at health centers in the 12 months prior to the survey. Postpartum hemorrhage (4.7%), obstructed/prolonged labor (3.0%), and postpartum sepsis (0.1%) account for the remaining half. The complications managed considered for the met need at health centers did not include hypertension/preeclampsia (though provide prereferral MgSO_4_ and antihypertensive drugs), ectopic pregnancy, and uterine rupture, which are managed at hospitals with CEmONC services.

### Relationship between changes in BIS over time and changes in service utilization of facility birth provision at the primary care level

**Fig. 3:**
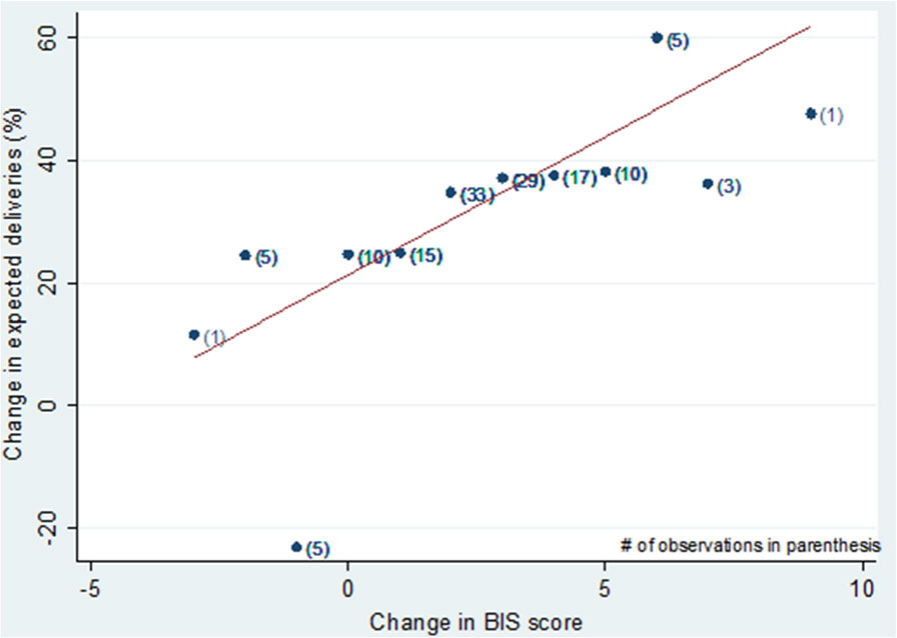
Association between change in health center delivery rates and change in BIS score

Improvements in the BIS score over the study period were significantly associated with improvements in health center delivery: for every unit increase in BIS score across time, there was a 4.5 percentage points increase in facility-based deliveries at health centers (Table 6 and Fig. 3). However, some health centers’ BIS score declined over time, mainly due to the decline in the availability of medical equipment and infection-prevention amenities.

**Table 6:** Fixed-effect model estimates of effect of BIS score on health center delivery propensity

Independent variable	Ordinary least-squares regression predicting health center delivery		
	Coefficient	(95% CI)	*p*-value
BIS score	4.5	(2.1, 6.9)	<.001
Constant	21.3	(13.5, 29.1)	<.001

### Relationship between changes in BIS score over time and the met need for BEmONC at the primary care level

**Fig. 4:**
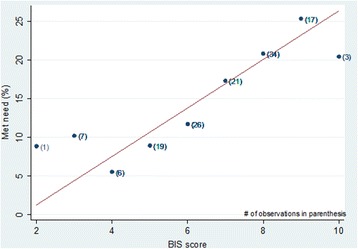
Cross-sectional association between the met need for BEmONC at health centers and BIS score

During the follow-up survey, facilities with a higher BIS score were associated with a higher met need for BEmONC (Table 7 and Fig. 4). On average, every unit higher BIS score of a facility was associated with 3.1 percentage points higher met need for BEmONC.

**Table 7:** Ordinary least square model estimates for effect of BIS score on BEmONC met need

Independent variable	Ordinary least-squares regression predicting the met need for BEmONC at health centers		
	Coefficient	(95% CI)	*p*-value
BIS score	3.1	(1.6, 4.6)	<.001
Constant	−5.0	(−15.5, 5.4)	.344

## Discussion

Our study is unique in reporting the effect of BIS on programmatic outcomes at the primary level of care. The variability in BIS across health facilities and time has demonstrated a significant dose–response association with the key program outcomes: for every unit increase in BIS score, there was a corresponding average increase of four to five facility-based delivery rates at the primary health care level. Moreover, a higher BIS score was associated with a higher met need for BEmONC.

This study showed that facility input and process indicators increased significantly from the baseline survey, demonstrating an increased level of readiness for emergencies and management of common obstetric and newborn complications in primary health care facilities. However, some basic interventions like neonatal resuscitation and the manual removal of the placenta did not change significantly, which might be due to the lack of specific technical skills. Accordingly, focused and more intense mentoring and supportive supervision for particular skills, such as neonatal resuscitation and the manual removal of the placenta, could be implemented to improve these interventions.

The mean number of skilled birth attendants (midwives, nurses, and health officers) and pharmacy and laboratory professionals working at health centers available at the time of the survey were in line with the minimum requirements [42].

The implementation strength of BEmONC is strongly associated with the improved availability and utilization of obstetric services in the intervention facilities, which ultimately will enhance the uptake of life-saving interventions to tackle the major causes of maternal and neonatal mortality in Ethiopia. This indicates that investment in the BEmONC initiative to upgrade primary level facilities is effective. As such, policymakers and program planners should make additional investments in improving the availability of critical inputs for the provision of BEmONC and closely monitor the process of service delivery at the primary health care level, to improve the utilization of maternal and newborn health services.

Conventionally, the availability of BEmONC signal functions at health facilities is used to monitor BEmONC programs [17]. The BIS can supplement and complement this by providing program input and process indicators that are essential for program managers to plan and monitor the provision of BEmONC services. Moreover, the BIS index predicts the BEmONC initiative outcomes well and has a good internal reliability. Thus, this would help to measure effort over time and gauge improvements of upgrading facilities for the provision of basic life-saving interventions and the effect of this effort on the utilization of maternal services outcomes at the primary level of care.

It should be noted that the BIS index would serve to monitor BEmONC implementation status of a health facility at a glance; but is not suitable for making a programmatic decision. If the BIS index score is at its maximum or near its maximum value then it can be concluded that all the components of the BEmONC interventions are being well implemented. However, if the BIS index score is not at the maximum or near the maximum value then individual items of the BIS index should be monitored to identify the areas with a gap.

There are several limitations with this study. First, the study used a nonexperimental program evaluation design; as such, the effect estimates could be confounded by unmeasured variables and the presence of possible selection bias. Second, the BIS index is the aggregation of 12 items. As a result, measurement error in one of the items would potentially introduce measurement error to the composite indicator. The observed association between BIS and the outcome variables would likely be inconsistent if the measurement error of the BIS is systematic.

Third, the change in data collection methods (paper-based during the baseline and mHealth-based during the follow-up) and different survey seasons between baseline and follow-up could bias the observed changes in the BIS index score and its components. However, these would be unlikely to affect the observed associations between the dependent and independent variables. Fourth, there is temporal ambiguity in the cross-sectional association between the met need for BEmONC and BIS index. Lastly, the BIS measure did not include all vital program input indicators. For example, although all health centers under study received post-training follow-up visits, the BIS index did not include whether any additional supervision and mentoring efforts were being provided as well as other supportive supervision efforts by hospitals and other partners, due to the problem of data verification as part of the BIS, which may have had a positive influence on the utilization of facility delivery and the met need for BEmONC.

We recommend further research on the quality of intrapartum care, the effect of BIS on the use of other maternal and newborn health services, equitable use of BEmONC services, and the cost of the BEmONC initiative. Provision of quality BEmONC services is an essential component for reducing maternal and newborn mortality rates [17]. As such, we recommend another study to investigate the effect of BIS on the obstetric complications case fatality rate, as well as on stillbirth and early neonatal death rates. Moreover, examining the effect of BIS on the utilization of other maternal and newborn health services is equally important. Improved uptake of BEmONC does not guarantee all women are using these services fairly; disadvantaged women might not access and use life-saving interventions. Disaggregated equity analysis of access to life-saving services would help health policy makers devise strategies to ensure equitable access to life-saving interventions for pregnant women who are beyond timely access to BEmONC and for poor women. Accordingly, we recommend other studies to examine the equitable use of BEmONC services by mothers. Furthermore, analyzing the cost of the BEmONC initiative is critical for policymakers and program planners, as an evidence base to prioritize BEmONC and improve maternal and newborn health at the primary health care level.

Finally, we used equal weights for the 12 items to construct the BIS index, which might be questionable. Thus, we would like to recommend further research to examine whether and how to give differential weights to different items measuring the BIS scores at the primary care level.

## Conclusion

The BEmONC initiative was effective in improving institutional deliveries and may have also improved the met need for BEmONC services. Potentially, the BIS index, including its components, could be used to monitor the implementation of BEmONC interventions.

## Additional files

Additional file 1: Survey dataset. This is survey data and data dictionary we used for the analysis. The first sheet contains the data variables and values and the second sheet contains variable definitions (data dictionary). (XLSX 289 kb)

Additional file 2: Survey Questionnaire. Survey questionnaire we used to collect information from facilities. (DOCX 149 kb)

## Abbreviations

BEmONC: Basic Emergency Obstetric and Newborn Care
BIS: BEmONC Implementation Strength
EmONC: Emergency Obstetric and Newborn Care
HIV: Human Immunodeficiency Virus
HSTP: Health Sector Transformation Plan
IV: Intravenous
LMIC: Low- and Middle-Income Countries
MMR: Maternal Mortality Ratio
NMR: Neonatal Mortality Rate
PHCU: Primary Health Care Unit
VDRL: Venereal Disease Research Laboratory test
