# Supplementary material for: The effect of implementation strength of basic emergency obstetric and newborn care (BEmONC) on facility deliveries and the met need for BEmONC at the primary health care level in Ethiopia
Source: BMC Pregnancy Childbirth. 2018 May 2;18:123. doi: 10.1186/s12884-018-1751-z (PMC5932776; doi:10.1186/s12884-018-1751-z)
Supplement: Supplementary file 2 — Survey Questionnaire. Survey questionnaire we used to collect information from facilities. (DOCX 149 kb) [file 12884_2018_1751_MOESM2_ESM.docx]

#### BEmONCOR_HC_Assessment

| Field | Question | Answer |
| --- | --- | --- |
| intronote | Measuring Implementation Strength of BEmONC Care: Health Center Assessment Tool JSI Research & Training Institute Inc., L10K Project |  |
| part1note | Part I: Health Facility Assessment |  |
| section1note | Section I: Facility Identification |  |
| q2 | Region? | \|  \| 1 \| Amhara \| \| --- \| --- \| --- \| \|  \| 2 \| Oromia \| \|  \| 3 \| SNNP \| \|  \| 4 \| Tigray \| |
| q3 | Zone? |  |
| q4 | Woreda? |  |
| q5 | Health center name? |  |
| q1 (required) | Date of visit |  |
| q7 (required) | Catchment population of the HC  Response constrained to: .>=1000 and .<=500000 |  |
| q104_hew (required) | How many health extension workers are in the catchment area?  Response constrained to: .>=1 and .<=30 |  |
| Distance to the nearest referral hospital | | |
| \|  \| q109_dis (required) \| \| --- \| --- \| | Distance to the nearest referral hospital  Record in kilo meter  Response constrained to: .>=1 and .<=300 |  |
| \|  \| q110_dis (required) \| \| --- \| --- \| | Distance to the nearest referral hospital with surgical care (CEmONC facility)  Record in kilo meter  Response constrained to: .>=1 and .<=300 |  |
| section2note | Section II: Functioning health facility |  |
| How many providers are currently working in this facility? | | |
| \|  \| q16 (required) \| \| --- \| --- \| | Midwives (all types)  Response constrained to: .>=0 and .<=10 |  |
| \|  \| q17 (required) \| \| --- \| --- \| | Health officer  Response constrained to: .>=0 and .<=10 |  |
| \|  \| q19 (required) \| \| --- \| --- \| | Nurse (all types)  Response constrained to: .>=0 and .<=20 |  |
| \|  \| q20 (required) \| \| --- \| --- \| | Pharmacy (all types)  Response constrained to: .>=0 and .<=10 |  |
| \|  \| q21 (required) \| \| --- \| --- \| | Laboratory (all types)  Response constrained to: .>=0 and .<=10 |  |
| How many providers are trained on BEmONC? | | |
| \|  \| q22 (required) \| \| --- \| --- \| | Midwives (all types)  Response constrained to: .>=0 and .<=10 |  |
| \|  \| q202_ho (required) \| \| --- \| --- \| | Health officer  Response constrained to: .>=0 and .<=10 |  |
| \|  \| q202_nurse (required) \| \| --- \| --- \| | Nurse (all types)  Response constrained to: .>=0 and .<=20 |  |
| How many providers are trained on BEmONC from L10K? | | |
| \|  \| q203_midwives (required) \| \| --- \| --- \| | Midwives (all types)  Response constrained to: .>=0 and .<=10 |  |
| \|  \| q203_ho (required) \| \| --- \| --- \| | Health officer  Response constrained to: .>=0 and .<=10 |  |
| \|  \| q203_nurse (required) \| \| --- \| --- \| | Nurse (all types)  Response constrained to: .>=0 and .<=20 |  |
| Of the providers currently working in the maternity unit, how many providers are on duty on the day of the visit? | | |
| \|  \| q204_midwives (required) \| \| --- \| --- \| | Midwives (all types)  Response constrained to: .>=0 and .<=10 |  |
| \|  \| q204_ho (required) \| \| --- \| --- \| | Health officer  Response constrained to: .>=0 and .<=10 |  |
| \|  \| q204_nurse (required) \| \| --- \| --- \| | Nurse (all types)  Response constrained to: .>=0 and .<=10 |  |
| \|  \| q204_phar (required) \| \| --- \| --- \| | Pharmacy (all types)  Response constrained to: .>=0 and .<=10 |  |
| \|  \| q204_lab (required) \| \| --- \| --- \| | Laboratory (all types)  Response constrained to: .>=0 and .<=10 |  |
| Are the following basic obstetric care services/ inputs available in the facility 24 hours a day? | | |
| \|  \| q205_antibiotics (required) \| \| --- \| --- \| | Parenteral antibiotics | \|  \| 1 \| Round the clock \| \| --- \| --- \| --- \| \|  \| 2 \| Day time only \| \|  \| 3 \| Other \| \|  \| 4 \| Not available \| |
| \|  \| q205_oxytocin (required) \| \| --- \| --- \| | IV Oxytocic drugs | \|  \| 1 \| Round the clock \| \| --- \| --- \| --- \| \|  \| 2 \| Day time only \| \|  \| 3 \| Other \| \|  \| 4 \| Not available \| |
| \|  \| q205_mgso4 (required) \| \| --- \| --- \| | Parenteral MgSO4 | \|  \| 1 \| Round the clock \| \| --- \| --- \| --- \| \|  \| 2 \| Day time only \| \|  \| 3 \| Other \| \|  \| 4 \| Not available \| |
| \|  \| q205_diazepam (required) \| \| --- \| --- \| | Parenteral diazepam | \|  \| 1 \| Round the clock \| \| --- \| --- \| --- \| \|  \| 2 \| Day time only \| \|  \| 3 \| Other \| \|  \| 4 \| Not available \| |
| \|  \| q205_placenta (required) \| \| --- \| --- \| | Manual removal of retained placenta | \|  \| 1 \| Round the clock \| \| --- \| --- \| --- \| \|  \| 2 \| Day time only \| \|  \| 3 \| Other \| \|  \| 4 \| Not available \| |
| \|  \| q205_retainedproducts (required) \| \| --- \| --- \| | Removal of retained products | \|  \| 1 \| Round the clock \| \| --- \| --- \| --- \| \|  \| 2 \| Day time only \| \|  \| 3 \| Other \| \|  \| 4 \| Not available \| |
| \|  \| q205_asbitrh (required) \| \| --- \| --- \| | Assisted vaginal delivery | \|  \| 1 \| Round the clock \| \| --- \| --- \| --- \| \|  \| 2 \| Day time only \| \|  \| 3 \| Other \| \|  \| 4 \| Not available \| |
| \|  \| q205_nresucitation (required) \| \| --- \| --- \| | Neonatal resuscitation with bag and mask | \|  \| 1 \| Round the clock \| \| --- \| --- \| --- \| \|  \| 2 \| Day time only \| \|  \| 3 \| Other \| \|  \| 4 \| Not available \| |
| \|  \| q25 (required) \| \| --- \| --- \| | Trained staff on duty to manage obstetric complications | \|  \| 1 \| Round the clock \| \| --- \| --- \| --- \| \|  \| 2 \| Day time only \| \|  \| 3 \| Other \| \|  \| 4 \| Not available \| |
| \|  \| q205_tscall (required) \| \| --- \| --- \| | Trained staff on call to manage obstetric complications | \|  \| 1 \| Round the clock \| \| --- \| --- \| --- \| \|  \| 2 \| Day time only \| \|  \| 3 \| Other \| \|  \| 4 \| Not available \| |
| \|  \| q64 (required) \| \| --- \| --- \| | Ambulance service | \|  \| 1 \| Round the clock \| \| --- \| --- \| --- \| \|  \| 2 \| Day time only \| \|  \| 3 \| Other \| \|  \| 4 \| Not available \| |
| \|  \| q67 (required) \| \| --- \| --- \| | Referral focal person | \|  \| 1 \| Round the clock \| \| --- \| --- \| --- \| \|  \| 2 \| Day time only \| \|  \| 3 \| Other \| \|  \| 4 \| Not available \| |
| \|  \| q74 (required) \| \| --- \| --- \| | Pharmacy service | \|  \| 1 \| Round the clock \| \| --- \| --- \| --- \| \|  \| 2 \| Day time only \| \|  \| 3 \| Other \| \|  \| 4 \| Not available \| |
| \|  \| q98 (required) \| \| --- \| --- \| | Laboratory service | \|  \| 1 \| Round the clock \| \| --- \| --- \| --- \| \|  \| 2 \| Day time only \| \|  \| 3 \| Other \| \|  \| 4 \| Not available \| |
| q37 (required) | Is the electric system currently functioning in the labour, delivery and post-partum rooms? | \|  \| 1 \| Yes \| \| --- \| --- \| --- \| \|  \| 0 \| No \| |
| q39 (required) | Is the water system currently functioning in the labour, delivery and post-partum rooms? | \|  \| 1 \| Yes \| \| --- \| --- \| --- \| \|  \| 0 \| No \| |
| Which of the following equipment and supplies are available and functional at this facility? | | |
| \|  \| q75 (required) \| \| --- \| --- \| | Sphygmomanometer | \|  \| 1 \| Yes \| \| --- \| --- \| --- \| \|  \| 0 \| No \| |
| \|  \| q78 (required) \| \| --- \| --- \| | Oxygen concentrator | \|  \| 1 \| Yes \| \| --- \| --- \| --- \| \|  \| 0 \| No \| |
| \|  \| q80 (required) \| \| --- \| --- \| | Surgical Gloves | \|  \| 1 \| Yes \| \| --- \| --- \| --- \| \|  \| 0 \| No \| |
| \|  \| q81 (required) \| \| --- \| --- \| | Vacuum extractor (sets) | \|  \| 1 \| Yes \| \| --- \| --- \| --- \| \|  \| 0 \| No \| |
| \|  \| q82 (required) \| \| --- \| --- \| | Suction machine | \|  \| 1 \| Yes \| \| --- \| --- \| --- \| \|  \| 0 \| No \| |
| \|  \| q86 (required) \| \| --- \| --- \| | Suction catheter | \|  \| 1 \| Yes \| \| --- \| --- \| --- \| \|  \| 0 \| No \| |
| \|  \| q84 (required) \| \| --- \| --- \| | Radiant heater | \|  \| 1 \| Yes \| \| --- \| --- \| --- \| \|  \| 0 \| No \| |
| \|  \| q85 (required) \| \| --- \| --- \| | Ambu bag & masks | \|  \| 1 \| Yes \| \| --- \| --- \| --- \| \|  \| 0 \| No \| |
| \|  \| q208_mva (required) \| \| --- \| --- \| | MVA set or E&C/ D&C set | \|  \| 1 \| Yes \| \| --- \| --- \| --- \| \|  \| 0 \| No \| |
| Which of the following drugs are available at this facility? | | |
| \|  \| q209_oxytocin (required) \| \| --- \| --- \| | IV uterotonics | \|  \| 1 \| Yes \| \| --- \| --- \| --- \| \|  \| 0 \| No \| |
| \|  \| q88 (required) \| \| --- \| --- \| | IV fluids | \|  \| 1 \| Yes \| \| --- \| --- \| --- \| \|  \| 0 \| No \| |
| \|  \| q89 (required) \| \| --- \| --- \| | Nifidipine | \|  \| 1 \| Yes \| \| --- \| --- \| --- \| \|  \| 0 \| No \| |
| \|  \| q90 (required) \| \| --- \| --- \| | Hydralazine | \|  \| 1 \| Yes \| \| --- \| --- \| --- \| \|  \| 0 \| No \| |
| \|  \| q91 (required) \| \| --- \| --- \| | Canula | \|  \| 1 \| Yes \| \| --- \| --- \| --- \| \|  \| 0 \| No \| |
| \|  \| q92 (required) \| \| --- \| --- \| | IV antibiotics | \|  \| 1 \| Yes \| \| --- \| --- \| --- \| \|  \| 0 \| No \| |
| \|  \| q94 (required) \| \| --- \| --- \| | IV MgSO4 | \|  \| 1 \| Yes \| \| --- \| --- \| --- \| \|  \| 0 \| No \| |
| \|  \| q95 (required) \| \| --- \| --- \| | IV Diazepam | \|  \| 1 \| Yes \| \| --- \| --- \| --- \| \|  \| 0 \| No \| |
| \|  \| q96 (required) \| \| --- \| --- \| | Calcium gluconate | \|  \| 1 \| Yes \| \| --- \| --- \| --- \| \|  \| 0 \| No \| |
| \|  \| q209_antipain (required) \| \| --- \| --- \| | IV analgesics | \|  \| 1 \| Yes \| \| --- \| --- \| --- \| \|  \| 0 \| No \| |
| Which of the following laboratory tests are available at the facility? | | |
| \|  \| q99 (required) \| \| --- \| --- \| | Hgb/HCT | \|  \| 1 \| Yes \| \| --- \| --- \| --- \| \|  \| 0 \| No \| |
| \|  \| q100 (required) \| \| --- \| --- \| | Blood Group | \|  \| 1 \| Yes \| \| --- \| --- \| --- \| \|  \| 0 \| No \| |
| \|  \| q101 (required) \| \| --- \| --- \| | U/A | \|  \| 1 \| Yes \| \| --- \| --- \| --- \| \|  \| 0 \| No \| |
| \|  \| q102 (required) \| \| --- \| --- \| | VDRL | \|  \| 1 \| Yes \| \| --- \| --- \| --- \| \|  \| 0 \| No \| |
| \|  \| q103 (required) \| \| --- \| --- \| | HIV test for PMTCT | \|  \| 1 \| Yes \| \| --- \| --- \| --- \| \|  \| 0 \| No \| |
| q118 (required) | Is maternity waiting area/ homes available in or around the facility? | \|  \| 1 \| Yes \| \| --- \| --- \| --- \| \|  \| 0 \| No \| |
| How many functional maternity beds are available? | | |
| \|  \| q121 (required) \| \| --- \| --- \| | Delivery couches  Response constrained to: .>=0 and .<=20 |  |
| \|  \| q122 (required) \| \| --- \| --- \| | 1st stage beds  Response constrained to: .>=0 and .<=10 |  |
| \|  \| q123 (required) \| \| --- \| --- \| | Post-partum beds  Response constrained to: .>=0 and .<=10 |  |
| q124 (required) | Is emergency cabinet available?  Observe for proper cabinet and availability of emergency drugs like IV fluids, Pitocin, canula, Diazepam/MgSO4, IV antibiotics and adrenalin | \|  \| 1 \| Yes \| \| --- \| --- \| --- \| \|  \| 0 \| No \| |
| q214 (required) | Is instrument set ready for assisted vaginal birth (vacuum extractor)?  Observe the availability of vacuum set and suction machine in labour room | \|  \| 1 \| Yes \| \| --- \| --- \| --- \| \|  \| 0 \| No \| |
| q128 (required) | Is a newborn corner available?  Observe the availability of ambu bag and masks, oxygen concentrator, radiant heater and suction machine | \|  \| 1 \| Yes \| \| --- \| --- \| --- \| \|  \| 0 \| No \| |
| q136 (required) | Is there light source for vaginal procedure in labor floor? | \|  \| 1 \| Yes \| \| --- \| --- \| --- \| \|  \| 0 \| No \| |
| Are the following conditions available in the maternity unit? | | |
| \|  \| q139 (required) \| \| --- \| --- \| | Waiting area for family or companion | \|  \| 1 \| Yes \| \| --- \| --- \| --- \| \|  \| 0 \| No \| |
| \|  \| q134 (required) \| \| --- \| --- \| | Enough physical space | \|  \| 1 \| Yes \| \| --- \| --- \| --- \| \|  \| 0 \| No \| |
| \|  \| q137 (required) \| \| --- \| --- \| | Good illumination and ventilation | \|  \| 1 \| Yes \| \| --- \| --- \| --- \| \|  \| 0 \| No \| |
| \|  \| q130 (required) \| \| --- \| --- \| | Easily washable delivery floor | \|  \| 1 \| Yes \| \| --- \| --- \| --- \| \|  \| 0 \| No \| |
| \|  \| q133 (required) \| \| --- \| --- \| | Delivery bed covered with washable plastic | \|  \| 1 \| Yes \| \| --- \| --- \| --- \| \|  \| 0 \| No \| |
| q218 (required) | Is toilet available for mothers? | \|  \| 1 \| Yes \| \| --- \| --- \| --- \| \|  \| 0 \| No \| |
| q219 (required) | Is there a shower in functioning condition that is available for mothers? | \|  \| 1 \| Yes \| \| --- \| --- \| --- \| \|  \| 0 \| No \| |
| Observe how infection prevention in the maternity unit (ANC, delivery and PNC) is practiced | | |
| \|  \| q104 (required) \| \| --- \| --- \| | Compound is clean | \|  \| 1 \| Yes \| \| --- \| --- \| --- \| \|  \| 0 \| No \| |
| \|  \| q105 (required) \| \| --- \| --- \| | Cleaning regularly done after attending birth | \|  \| 1 \| Yes \| \| --- \| --- \| --- \| \|  \| 0 \| No \| |
| \|  \| q106 (required) \| \| --- \| --- \| | Soap available at all sinks in the ANC, labor and postpartum floor | \|  \| 1 \| Yes \| \| --- \| --- \| --- \| \|  \| 0 \| No \| |
| \|  \| q107 (required) \| \| --- \| --- \| | Housekeeping staff has personal protective barriers | \|  \| 1 \| Yes \| \| --- \| --- \| --- \| \|  \| 0 \| No \| |
| \|  \| q108 (required) \| \| --- \| --- \| | Disinfectant and cleaning solutions area available | \|  \| 1 \| Yes \| \| --- \| --- \| --- \| \|  \| 0 \| No \| |
| \|  \| q109 (required) \| \| --- \| --- \| | Disinfectant solution prepared and used as per standard | \|  \| 1 \| Yes \| \| --- \| --- \| --- \| \|  \| 0 \| No \| |
| \|  \| q110 (required) \| \| --- \| --- \| | Instrument processing area and sets are available | \|  \| 1 \| Yes \| \| --- \| --- \| --- \| \|  \| 0 \| No \| |
| \|  \| q111 (required) \| \| --- \| --- \| | Puncture proof container for sharps is available | \|  \| 1 \| Yes \| \| --- \| --- \| --- \| \|  \| 0 \| No \| |
| \|  \| q112 (required) \| \| --- \| --- \| | Providers practice hand washing | \|  \| 1 \| Yes \| \| --- \| --- \| --- \| \|  \| 0 \| No \| |
| \|  \| q113 (required) \| \| --- \| --- \| | Quality mechanism for standard of sterilization  autoclave calibrated regularly , steam sterilizer function indicator monitored regularly | \|  \| 1 \| Yes \| \| --- \| --- \| --- \| \|  \| 0 \| No \| |
| \|  \| q114 (required) \| \| --- \| --- \| | Guideline and job aid for IP is available | \|  \| 1 \| Yes \| \| --- \| --- \| --- \| \|  \| 0 \| No \| |
| \|  \| q115 (required) \| \| --- \| --- \| | Waste disposal system in place (Leak proof containers, waste is sorted, incinerator, placenta pit) | \|  \| 1 \| Yes \| \| --- \| --- \| --- \| \|  \| 0 \| No \| |
| \|  \| q116 (required) \| \| --- \| --- \| | Staff consistently uses personal protective barrier (PPB) | \|  \| 1 \| Yes \| \| --- \| --- \| --- \| \|  \| 0 \| No \| |
| section3note | Section III: Effective referral |  |
| q301 (required) | Does this facility have a functional ambulance or other vehicle for emergency transportation? | \|  \| 1 \| Yes \| \| --- \| --- \| --- \| \|  \| 0 \| No \| |
| q302 (required) | Does this facility have access to an ambulance or other vehicle for emergency? | \|  \| 1 \| Yes \| \| --- \| --- \| --- \| \|  \| 0 \| No \| |
| q303 (required) | Is the ambulance located in your facility premises?  Question relevant when: ${q302} = '1' | \|  \| 1 \| Yes \| \| --- \| --- \| --- \| \|  \| 0 \| No \| |
| q304 (required) | If ambulance is not always available, how long does it take to arrange it?  Record in minutes  Question relevant when: ${q303} = '0' |  |
| q305 (required) | Does this facility have a functioning land line telephone that is available to call outside at all times? | \|  \| 1 \| Yes \| \| --- \| --- \| --- \| \|  \| 0 \| No \| |
| q306 (required) | Does this facility have a functioning cellular telephone or a private cellular phone? | \|  \| 1 \| Yes \| \| --- \| --- \| --- \| \|  \| 0 \| No \| |
| q40 (required) | Is telephone available in the maternity area for two way communication? | \|  \| 1 \| Yes \| \| --- \| --- \| --- \| \|  \| 0 \| No \| |
| q65 (required) | Does the community have direct access to an ambulance or to a direct call system? | \|  \| 1 \| Yes \| \| --- \| --- \| --- \| \|  \| 0 \| No \| |
| q309 (required) | Is a referral focal person/liaison officer available at least for working days? | \|  \| 1 \| Yes \| \| --- \| --- \| --- \| \|  \| 0 \| No \| |
| q310 (required) | Is there a unit that coordinates referrals (office, office materials like communication methods-telephone, email etc)  Record observation | \|  \| 1 \| Yes \| \| --- \| --- \| --- \| \|  \| 0 \| No \| |
| q311 (required) | Are standard protocols (for who to refer, when and where) available?  Record observation | \|  \| 1 \| Yes \| \| --- \| --- \| --- \| \|  \| 0 \| No \| |
| q312 (required) | If yes, are providers oriented on their use?  Question relevant when: ${q311} = '1' | \|  \| 1 \| Yes \| \| --- \| --- \| --- \| \|  \| 0 \| No \| |
| Does the HC have standardized referral slips and registers in place? | | |
| \|  \| q313a (required) \| \| --- \| --- \| | Referral slip | \|  \| 1 \| Yes \| \| --- \| --- \| --- \| \|  \| 0 \| No \| |
| \|  \| q313b (required) \| \| --- \| --- \| | Referral out register | \|  \| 1 \| Yes \| \| --- \| --- \| --- \| \|  \| 0 \| No \| |
| \|  \| q313c (required) \| \| --- \| --- \| | Referral in register | \|  \| 1 \| Yes \| \| --- \| --- \| --- \| \|  \| 0 \| No \| |
| \|  \| q313d (required) \| \| --- \| --- \| | Service directory | \|  \| 1 \| Yes \| \| --- \| --- \| --- \| \|  \| 0 \| No \| |
| Do the HPs have standardized referral slips and referral-out registers in place? | | |
| \|  \| q314a (required) \| \| --- \| --- \| | Referral slip | \|  \| 1 \| Yes \| \| --- \| --- \| --- \| \|  \| 0 \| No \| |
| \|  \| q314b (required) \| \| --- \| --- \| | Referral out register | \|  \| 1 \| Yes \| \| --- \| --- \| --- \| \|  \| 0 \| No \| |
| q315 (required) | Are standard protocols (for who to refer, when and where) available at HPs? | \|  \| 1 \| Yes \| \| --- \| --- \| --- \| \|  \| 0 \| No \| |
| q316 (required) | If yes, are HEWs oriented on their use?  Question relevant when: ${q315} = '1' | \|  \| 1 \| Yes \| \| --- \| --- \| --- \| \|  \| 0 \| No \| |
| How often do the HEWs adhere to aspects of referral protocols while referring MNH cases to HC? | | |
| \|  \| q317a (required) \| \| --- \| --- \| | Sending with referral slip | \|  \| 1 \| Always \| \| --- \| --- \| --- \| \|  \| 2 \| Sometimes \| \|  \| 3 \| Rarely \| \|  \| 4 \| Never \| |
| \|  \| q317b (required) \| \| --- \| --- \| | Escorting & care during transfer | \|  \| 1 \| Always \| \| --- \| --- \| --- \| \|  \| 2 \| Sometimes \| \|  \| 3 \| Rarely \| \|  \| 4 \| Never \| |
| \|  \| q317c (required) \| \| --- \| --- \| | Arrange and Used ambulance | \|  \| 1 \| Always \| \| --- \| --- \| --- \| \|  \| 2 \| Sometimes \| \|  \| 3 \| Rarely \| \|  \| 4 \| Never \| |
| \|  \| q317d (required) \| \| --- \| --- \| | Pre-referral management | \|  \| 1 \| Always \| \| --- \| --- \| --- \| \|  \| 2 \| Sometimes \| \|  \| 3 \| Rarely \| \|  \| 4 \| Never \| |
| \|  \| q317e (required) \| \| --- \| --- \| | Received feedback | \|  \| 1 \| Always \| \| --- \| --- \| --- \| \|  \| 2 \| Sometimes \| \|  \| 3 \| Rarely \| \|  \| 4 \| Never \| |
| q318 (required) | Is there a system for this facility to receive a feedback from the receiving facility/referral hospital? | \|  \| 1 \| Yes \| \| --- \| --- \| --- \| \|  \| 0 \| No \| |
| q319 (required) | If so, which system?  Circle all that apply  Question relevant when: ${q318} = '1' | \|  \| 1 \| Verbal \| \| --- \| --- \| --- \| \|  \| 2 \| Section of referral form filled out and sent back \| \|  \| 3 \| Separate counter-referral form \| \|  \| 4 \| Blank slip of paper \| \|  \| 5 \| Telephone \| \|  \| 88 \| Other \| |
| section4note | Section IV: Provision of care |  |
| anc | ANC observation |  |
| q140 (required) | Is privacy maintained in the ANC room?  (Area for counseling and examination, curtain or partition on examination areas...) | \|  \| 1 \| Yes \| \| --- \| --- \| --- \| \|  \| 0 \| No \| |
| Counseling provided on the following topics | | |
| \|  \| q402a (required) \| \| --- \| --- \| | Nutrition | \|  \| 1 \| Yes \| \| --- \| --- \| --- \| \|  \| 0 \| No \| |
| \|  \| q402b (required) \| \| --- \| --- \| | Birth preparedness complication readiness | \|  \| 1 \| Yes \| \| --- \| --- \| --- \| \|  \| 0 \| No \| |
| \|  \| q402c (required) \| \| --- \| --- \| | Child birthing | \|  \| 1 \| Yes \| \| --- \| --- \| --- \| \|  \| 0 \| No \| |
| \|  \| q402d (required) \| \| --- \| --- \| | PMTCT | \|  \| 1 \| Yes \| \| --- \| --- \| --- \| \|  \| 0 \| No \| |
| \|  \| q402e (required) \| \| --- \| --- \| | Syphilis | \|  \| 1 \| Yes \| \| --- \| --- \| --- \| \|  \| 0 \| No \| |
| \|  \| q402f (required) \| \| --- \| --- \| | Other STIs | \|  \| 1 \| Yes \| \| --- \| --- \| --- \| \|  \| 0 \| No \| |
| \|  \| q402g (required) \| \| --- \| --- \| | Malaria | \|  \| 1 \| Yes \| \| --- \| --- \| --- \| \|  \| 0 \| No \| |
| Is birth preparedness and complication readiness plan developed jointly? | | |
| \|  \| q403a (required) \| \| --- \| --- \| | Place of birth | \|  \| 1 \| Yes \| \| --- \| --- \| --- \| \|  \| 0 \| No \| |
| \|  \| q403b (required) \| \| --- \| --- \| | Emergency transportation | \|  \| 1 \| Yes \| \| --- \| --- \| --- \| \|  \| 0 \| No \| |
| \|  \| q403c (required) \| \| --- \| --- \| | Money | \|  \| 1 \| Yes \| \| --- \| --- \| --- \| \|  \| 0 \| No \| |
| \|  \| q403d (required) \| \| --- \| --- \| | Supplies needed for birth | \|  \| 1 \| Yes \| \| --- \| --- \| --- \| \|  \| 0 \| No \| |
| \|  \| q403e (required) \| \| --- \| --- \| | Support person | \|  \| 1 \| Yes \| \| --- \| --- \| --- \| \|  \| 0 \| No \| |
| \|  \| q403f (required) \| \| --- \| --- \| | Potential blood donor | \|  \| 1 \| Yes \| \| --- \| --- \| --- \| \|  \| 0 \| No \| |
| \|  \| q403g (required) \| \| --- \| --- \| | Danger signs in labour | \|  \| 1 \| Yes \| \| --- \| --- \| --- \| \|  \| 0 \| No \| |
| delandpnc | Delivery, PNC and newborn care (Observation and interview) |  |
| q404 (required) | Observe for delivery room privacy, that is a room with partition or curtains and client is not exposed unduly | \|  \| 1 \| Privacy kept \| \| --- \| --- \| --- \| \|  \| 2 \| Privacy not kept \| |
| Is client centered care provided? | | |
| \|  \| q159 (required) \| \| --- \| --- \| | Mothers allowed to choose position | \|  \| 1 \| Yes \| \| --- \| --- \| --- \| \|  \| 0 \| No \| |
| \|  \| q160 (required) \| \| --- \| --- \| | Family member allowed to companion | \|  \| 1 \| Yes \| \| --- \| --- \| --- \| \|  \| 0 \| No \| |
| \|  \| q161 (required) \| \| --- \| --- \| | Cultural practices allowed (e.g. coffee, etc) | \|  \| 1 \| Yes \| \| --- \| --- \| --- \| \|  \| 0 \| No \| |
| q162 (required) | The provider receives and treats the laboring mother cordially, and conducts a quick check at the first contact | \|  \| 1 \| Yes \| \| --- \| --- \| --- \| \|  \| 0 \| No \| |
| Observe for items prepared to attended birth. If observation is not applicable please ask: Which items did you prepare during the last birth that you attended. | | |
| \|  \| q407a (required) \| \| --- \| --- \| | Disinfectant | \|  \| 1 \| Yes \| \| --- \| --- \| --- \| \|  \| 0 \| No \| |
| \|  \| q407b (required) \| \| --- \| --- \| | Gloves | \|  \| 1 \| Yes \| \| --- \| --- \| --- \| \|  \| 0 \| No \| |
| \|  \| q407c (required) \| \| --- \| --- \| | Gauze | \|  \| 1 \| Yes \| \| --- \| --- \| --- \| \|  \| 0 \| No \| |
| \|  \| q407d (required) \| \| --- \| --- \| | Emergency drugs including uterotonic drug | \|  \| 1 \| Yes \| \| --- \| --- \| --- \| \|  \| 0 \| No \| |
| \|  \| q407e (required) \| \| --- \| --- \| | Clean cloths | \|  \| 1 \| Yes \| \| --- \| --- \| --- \| \|  \| 0 \| No \| |
| \|  \| q407f (required) \| \| --- \| --- \| | Sterile blade/forceps | \|  \| 1 \| Yes \| \| --- \| --- \| --- \| \|  \| 0 \| No \| |
| \|  \| q407g (required) \| \| --- \| --- \| | Cord tie | \|  \| 1 \| Yes \| \| --- \| --- \| --- \| \|  \| 0 \| No \| |
| \|  \| q407h (required) \| \| --- \| --- \| | Soap | \|  \| 1 \| Yes \| \| --- \| --- \| --- \| \|  \| 0 \| No \| |
| \|  \| q407i (required) \| \| --- \| --- \| | Eye ointment | \|  \| 1 \| Yes \| \| --- \| --- \| --- \| \|  \| 0 \| No \| |
| \|  \| q407j (required) \| \| --- \| --- \| | Sufficient sterilized delivery sets | \|  \| 1 \| Yes \| \| --- \| --- \| --- \| \|  \| 0 \| No \| |
| \|  \| q407k (required) \| \| --- \| --- \| | Vacuum extractor | \|  \| 1 \| Yes \| \| --- \| --- \| --- \| \|  \| 0 \| No \| |
| \|  \| q407l (required) \| \| --- \| --- \| | Newborn resuscitation set | \|  \| 1 \| Yes \| \| --- \| --- \| --- \| \|  \| 0 \| No \| |
| q408 (required) | Observe the practice of third stage of active management (Pitocin , CCT and massage) If observation is not applicable ask: At the last birth attended did you administer prophylactic uterotonics, uterine massage and controlled cord traction for the prevention of PPH?  Circle all that apply | \|  \| 1 \| Administered prophylactic uterotonics \| \| --- \| --- \| --- \| \|  \| 2 \| Uterine massage \| \|  \| 3 \| Controlled Cord Traction (CCT) \| |
| q409 (required) | Does this facility routinely administer Oxytocin injection immediately after birth to all women for the prevention of post-partum haemorrhage? | \|  \| 1 \| Yes \| \| --- \| --- \| --- \| \|  \| 0 \| No \| |
| q410 (required) | At the last birth attended, what care did you give the newborn? Prove: What else?  Circle all that apply | \|  \| 1 \| Dry the baby and wrap with dry clothes \| \| --- \| --- \| --- \| \|  \| 2 \| Keep with the mother in skin-to-skin contact \| \|  \| 3 \| Apply Tetracycline eye ointment \| \|  \| 4 \| Give immunization \| \|  \| 5 \| Weigh the baby \| \|  \| 6 \| Vitamin K \| \|  \| 88 \| Other \| |
| q411 (required) | Has a breech delivery been performed in the last 3 months? | \|  \| 1 \| Yes \| \| --- \| --- \| --- \| \|  \| 0 \| No \| |
| q412 (required) | If a breech delivery was NOT performed in the last 3 months, why?  Circle all that apply  Question relevant when: ${q411} = '0' | \|  \| 1 \| availability of human resources \| \| --- \| --- \| --- \| \|  \| 2 \| training issues \| \|  \| 3 \| supplies/equipment/drugs \| \|  \| 4 \| management issues \| \|  \| 5 \| policy issues \| \|  \| 6 \| no indication \| \|  \| 88 \| Other \| |
| sfunctions | Performance of signal functions |  |
| Which of the following signal functions performed in this facility in the last 3 | | |
| \|  \| q146 (required) \| \| --- \| --- \| | Use of IV antibiotics | \|  \| 1 \| Yes \| \| --- \| --- \| --- \| \|  \| 0 \| No \| |
| \|  \| q147 (required) \| \| --- \| --- \| | Use of uterotonics | \|  \| 1 \| Yes \| \| --- \| --- \| --- \| \|  \| 0 \| No \| |
| \|  \| q148 (required) \| \| --- \| --- \| | Use of Magso4/Diazepam for treatment of PEE | \|  \| 1 \| Yes \| \| --- \| --- \| --- \| \|  \| 0 \| No \| |
| \|  \| q149 (required) \| \| --- \| --- \| | Removal of retained products of conception | \|  \| 1 \| Yes \| \| --- \| --- \| --- \| \|  \| 0 \| No \| |
| \|  \| q150 (required) \| \| --- \| --- \| | Manual removal of placenta | \|  \| 1 \| Yes \| \| --- \| --- \| --- \| \|  \| 0 \| No \| |
| \|  \| q151 (required) \| \| --- \| --- \| | Assisted vaginal birth | \|  \| 1 \| Yes \| \| --- \| --- \| --- \| \|  \| 0 \| No \| |
| \|  \| q152 (required) \| \| --- \| --- \| | Neonatal resuscitation | \|  \| 1 \| Yes \| \| --- \| --- \| --- \| \|  \| 0 \| No \| |
| Which of the following signal functions performed in this facility in the last 12 months? | | |
| \|  \| q413a_12 (required) \| \| --- \| --- \| | Use of IV antibiotics | \|  \| 1 \| Yes \| \| --- \| --- \| --- \| \|  \| 0 \| No \| |
| \|  \| q413b_12 (required) \| \| --- \| --- \| | Use of uterotonics | \|  \| 1 \| Yes \| \| --- \| --- \| --- \| \|  \| 0 \| No \| |
| \|  \| q413c_12 (required) \| \| --- \| --- \| | Use of Magso4/Diazepam for treatment of PEE | \|  \| 1 \| Yes \| \| --- \| --- \| --- \| \|  \| 0 \| No \| |
| \|  \| q413d_12 (required) \| \| --- \| --- \| | Removal of retained products of conception | \|  \| 1 \| Yes \| \| --- \| --- \| --- \| \|  \| 0 \| No \| |
| \|  \| q413e_12 (required) \| \| --- \| --- \| | Manual removal of placenta | \|  \| 1 \| Yes \| \| --- \| --- \| --- \| \|  \| 0 \| No \| |
| \|  \| q413f_12 (required) \| \| --- \| --- \| | Assisted vaginal birth | \|  \| 1 \| Yes \| \| --- \| --- \| --- \| \|  \| 0 \| No \| |
| \|  \| q413g_12 (required) \| \| --- \| --- \| | Neonatal resuscitation | \|  \| 1 \| Yes \| \| --- \| --- \| --- \| \|  \| 0 \| No \| |
| q414 (required) | Which drug did you use to treat pre-eclampsia/eclampsia? | \|  \| 1 \| MgSO4 \| \| --- \| --- \| --- \| \|  \| 2 \| Diazepam \| \|  \| 3 \| Both \| |
| Reasons for missing | | |
| \|  \| q413a_reasons (required) \| \| --- \| --- \| | Use of IV antibiotics  Question relevant when: ${q146} = '0' or ${q413a_12} = '0' | \|  \| 1 \| no case \| \| --- \| --- \| --- \| \|  \| 2 \| no supply \| \|  \| 3 \| no trained provider \| |
| \|  \| q413b_reasons (required) \| \| --- \| --- \| | Use of uterotonics  Question relevant when: ${q147} = '0' or ${q413b_12} = '0' | \|  \| 1 \| no case \| \| --- \| --- \| --- \| \|  \| 2 \| no supply \| \|  \| 3 \| no trained provider \| |
| \|  \| q413c_reasons (required) \| \| --- \| --- \| | Use of Magso4/Diazepam for treatment of PEE  Question relevant when: ${q148} = '0' or ${q413c_12} = '0' | \|  \| 1 \| no case \| \| --- \| --- \| --- \| \|  \| 2 \| no supply \| \|  \| 3 \| no trained provider \| |
| \|  \| q413d_reasons (required) \| \| --- \| --- \| | Removal of retained products of conception  Question relevant when: ${q149} = '0' or ${q413d_12} = '0' | \|  \| 1 \| no case \| \| --- \| --- \| --- \| \|  \| 2 \| no supply \| \|  \| 3 \| no trained provider \| |
| \|  \| q413e_reasons (required) \| \| --- \| --- \| | Manual removal of placenta  Question relevant when: ${q150} = '0' or ${q413e_12} = '0' | \|  \| 1 \| no case \| \| --- \| --- \| --- \| \|  \| 2 \| no supply \| \|  \| 3 \| no trained provider \| |
| \|  \| q413f_reasons (required) \| \| --- \| --- \| | Assisted vaginal birth  Question relevant when: ${q151} = '0' or ${q413f_12} = '0' | \|  \| 1 \| no case \| \| --- \| --- \| --- \| \|  \| 2 \| no supply \| \|  \| 3 \| no trained provider \| |
| \|  \| q413g_reasons (required) \| \| --- \| --- \| | Neonatal resuscitation  Question relevant when: ${q152} = '0' or ${q413g_12} = '0' | \|  \| 1 \| no case \| \| --- \| --- \| --- \| \|  \| 2 \| no supply \| \|  \| 3 \| no trained provider \| |
| On the day of the visit, is the health center ready to perform all BEmONC signal functions? | | |
| \|  \| q415a (required) \| \| --- \| --- \| | Trained provider available to provide signal functions | \|  \| 1 \| Yes \| \| --- \| --- \| --- \| \|  \| 0 \| No \| |
| \|  \| q415b (required) \| \| --- \| --- \| | All dugs available (IV antibiotics, MgSO4, uterotonics) to provide signal functions | \|  \| 1 \| Yes \| \| --- \| --- \| --- \| \|  \| 0 \| No \| |
| \|  \| q415c (required) \| \| --- \| --- \| | All equipment available to perform signal functions | \|  \| 1 \| Yes \| \| --- \| --- \| --- \| \|  \| 0 \| No \| |
| section5note | Section V: Supportive supervision |  |
| q501 (required) | When was the last time this facility received a supervision visit from the higher level (WorHO, ZHD, RHB, MOH or L10K)? | \|  \| 1 \| This month \| \| --- \| --- \| --- \| \|  \| 2 \| In the last 3 months \| \|  \| 3 \| More than 3 months ago \| \|  \| 99 \| Don’t know \| |
| q502 (required) | How many supportive supervision visits has this facility provided to cluster HPs in the last 3 months?  Response constrained to: .>=0 and .<=50 |  |
| q503 (required) | How many supportive supervision visits have you received in the last 3 months from WorHO, ZHD, RHB or MOH?  Question relevant when: ${q501} = '1' or ${q501} = '2'  Response constrained to: .>=0 and .<=10 |  |
| q504 (required) | How many review meetings have been held within PHCUs (HEWs and Health Center staffs) in the last 3 months?  Response constrained to: .>=0 and .<=10 |  |
| q505 (required) | How many on-site mentoring visits have been received from L10K in the last 12 months?  Response constrained to: .>=0 and .<=5 |  |
| q506 (required) | How many follow-up visits have been received from L10K in the last 12 months?  Response constrained to: .>=0 and .<=10 |  |
| part2note | Part II: BEmONC Trained Provider Knowledge and Competency  Now I am going to ask you about your knowledge and application of BEmONC skills learnt during the BEmONC training & mentoring |  |
| consent | Are you willing to participate? | \|  \| 1 \| Yes \| \| --- \| --- \| --- \| \|  \| 0 \| No \| |
| consent2  Group relevant when: ${consent} = '1' | | |
| \|  \| q601 (required) \| \| --- \| --- \| | What is your professional classification? | \|  \| 1 \| Health officer \| \| --- \| --- \| --- \| \|  \| 2 \| Midwife \| \|  \| 3 \| Nurse \| \|  \| 88 \| Other \| |
| \|  \| q601_oth (required) \| \| --- \| --- \| | Please specify other category  Question relevant when: ${q601} = '88' |  |
| \|  \| q602 (required) \| \| --- \| --- \| | How many years has it been since you received your professional qualification?  Please record in completed years  Response constrained to: .>=0 and .<=30 |  |
| \|  \| q603 (required) \| \| --- \| --- \| | How many deliveries did you attend last month?  Response constrained to: .>=0 and .<=60 |  |
| \|  \| consent2 > Have you applied the following skills in the last 3 months? \| \| --- \| --- \| | | |
| \|  \|  \| q604a (required) \| \| --- \| --- \| --- \| | Assisting normal deliver | \|  \| 1 \| Yes \| \| --- \| --- \| --- \| \|  \| 0 \| No \| |
| \|  \|  \| q604b (required) \| \| --- \| --- \| --- \| | Administration of Oxytocin | \|  \| 1 \| Yes \| \| --- \| --- \| --- \| \|  \| 0 \| No \| |
| \|  \|  \| q604c (required) \| \| --- \| --- \| --- \| | Administration of antibiotics | \|  \| 1 \| Yes \| \| --- \| --- \| --- \| \|  \| 0 \| No \| |
| \|  \|  \| q604d (required) \| \| --- \| --- \| --- \| | MgSO4/Diazepam administration | \|  \| 1 \| Yes \| \| --- \| --- \| --- \| \|  \| 0 \| No \| |
| \|  \|  \| q604e (required) \| \| --- \| --- \| --- \| | Removal of retained products | \|  \| 1 \| Yes \| \| --- \| --- \| --- \| \|  \| 0 \| No \| |
| \|  \|  \| q604f (required) \| \| --- \| --- \| --- \| | Manual removal of retained placenta | \|  \| 1 \| Yes \| \| --- \| --- \| --- \| \|  \| 0 \| No \| |
| \|  \|  \| q604g (required) \| \| --- \| --- \| --- \| | Vacuum extraction | \|  \| 1 \| Yes \| \| --- \| --- \| --- \| \|  \| 0 \| No \| |
| \|  \|  \| q604h (required) \| \| --- \| --- \| --- \| | Assisting breech delivery | \|  \| 1 \| Yes \| \| --- \| --- \| --- \| \|  \| 0 \| No \| |
| \|  \|  \| q604i (required) \| \| --- \| --- \| --- \| | Manual Vacuum Aspiration | \|  \| 1 \| Yes \| \| --- \| --- \| --- \| \|  \| 0 \| No \| |
| \|  \|  \| q604j (required) \| \| --- \| --- \| --- \| | AMTSL | \|  \| 1 \| Yes \| \| --- \| --- \| --- \| \|  \| 0 \| No \| |
| \|  \|  \| q604k (required) \| \| --- \| --- \| --- \| | Bi-manual compression of the uterus | \|  \| 1 \| Yes \| \| --- \| --- \| --- \| \|  \| 0 \| No \| |
| \|  \|  \| q604l (required) \| \| --- \| --- \| --- \| | Abdominal aortic compression | \|  \| 1 \| Yes \| \| --- \| --- \| --- \| \|  \| 0 \| No \| |
| \|  \|  \| q604m (required) \| \| --- \| --- \| --- \| | Episiotomy & Tear repair | \|  \| 1 \| Yes \| \| --- \| --- \| --- \| \|  \| 0 \| No \| |
| \|  \|  \| q604n (required) \| \| --- \| --- \| --- \| | Newborn resuscitation | \|  \| 1 \| Yes \| \| --- \| --- \| --- \| \|  \| 0 \| No \| |
| \|  \|  \| q604o (required) \| \| --- \| --- \| --- \| | Partograph use | \|  \| 1 \| Yes \| \| --- \| --- \| --- \| \|  \| 0 \| No \| |
| \|  \| consent2 > Reasons for missing \| \| --- \| --- \| | | |
| \|  \|  \| q604a_reasons (required) \| \| --- \| --- \| --- \| | Assisting normal deliver  Question relevant when: ${q604a} = '0' | \|  \| 1 \| No cases \| \| --- \| --- \| --- \| \|  \| 2 \| no supply \| \|  \| 3 \| Afraid of doing it \| \|  \| 4 \| Other \| |
| \|  \|  \| q604b_reasons (required) \| \| --- \| --- \| --- \| | Administration of Oxytocin  Question relevant when: ${q604b} = '0' | \|  \| 1 \| No cases \| \| --- \| --- \| --- \| \|  \| 2 \| no supply \| \|  \| 3 \| Afraid of doing it \| \|  \| 4 \| Other \| |
| \|  \|  \| q604c_reasons (required) \| \| --- \| --- \| --- \| | Administration of antibiotics  Question relevant when: ${q604c} = '0' | \|  \| 1 \| No cases \| \| --- \| --- \| --- \| \|  \| 2 \| no supply \| \|  \| 3 \| Afraid of doing it \| \|  \| 4 \| Other \| |
| \|  \|  \| q604d_reasons (required) \| \| --- \| --- \| --- \| | MgSO4/Diazepam administration  Question relevant when: ${q604d} = '0' | \|  \| 1 \| No cases \| \| --- \| --- \| --- \| \|  \| 2 \| no supply \| \|  \| 3 \| Afraid of doing it \| \|  \| 4 \| Other \| |
| \|  \|  \| q604e_reasons (required) \| \| --- \| --- \| --- \| | Removal of retained products  Question relevant when: ${q604e} = '0' | \|  \| 1 \| No cases \| \| --- \| --- \| --- \| \|  \| 2 \| no supply \| \|  \| 3 \| Afraid of doing it \| \|  \| 4 \| Other \| |
| \|  \|  \| q604f_reasons (required) \| \| --- \| --- \| --- \| | Manual removal of retained placenta  Question relevant when: ${q604f} = '0' | \|  \| 1 \| No cases \| \| --- \| --- \| --- \| \|  \| 2 \| no supply \| \|  \| 3 \| Afraid of doing it \| \|  \| 4 \| Other \| |
| \|  \|  \| q604g_reasons (required) \| \| --- \| --- \| --- \| | Vacuum extraction  Question relevant when: ${q604g} = '0' | \|  \| 1 \| No cases \| \| --- \| --- \| --- \| \|  \| 2 \| no supply \| \|  \| 3 \| Afraid of doing it \| \|  \| 4 \| Other \| |
| \|  \|  \| q604h_reasons (required) \| \| --- \| --- \| --- \| | Assisting breech delivery  Question relevant when: ${q604h} = '0' | \|  \| 1 \| No cases \| \| --- \| --- \| --- \| \|  \| 2 \| no supply \| \|  \| 3 \| Afraid of doing it \| \|  \| 4 \| Other \| |
| \|  \|  \| q604i_reasons (required) \| \| --- \| --- \| --- \| | Manual Vacuum Aspiration  Question relevant when: ${q604i} = '0' | \|  \| 1 \| No cases \| \| --- \| --- \| --- \| \|  \| 2 \| no supply \| \|  \| 3 \| Afraid of doing it \| \|  \| 4 \| Other \| |
| \|  \|  \| q604j_reasons (required) \| \| --- \| --- \| --- \| | AMTSL  Question relevant when: ${q604j} = '0' | \|  \| 1 \| No cases \| \| --- \| --- \| --- \| \|  \| 2 \| no supply \| \|  \| 3 \| Afraid of doing it \| \|  \| 4 \| Other \| |
| \|  \|  \| q604k_reasons (required) \| \| --- \| --- \| --- \| | Bi-manual compression of the uterus  Question relevant when: ${q604k} = '0' | \|  \| 1 \| No cases \| \| --- \| --- \| --- \| \|  \| 2 \| no supply \| \|  \| 3 \| Afraid of doing it \| \|  \| 4 \| Other \| |
| \|  \|  \| q604l_reasons (required) \| \| --- \| --- \| --- \| | Abdominal aortic compression  Question relevant when: ${q604l} = '0' | \|  \| 1 \| No cases \| \| --- \| --- \| --- \| \|  \| 2 \| no supply \| \|  \| 3 \| Afraid of doing it \| \|  \| 4 \| Other \| |
| \|  \|  \| q604m_reasons (required) \| \| --- \| --- \| --- \| | Episiotomy & Tear repair  Question relevant when: ${q604m} = '0' | \|  \| 1 \| No cases \| \| --- \| --- \| --- \| \|  \| 2 \| no supply \| \|  \| 3 \| Afraid of doing it \| \|  \| 4 \| Other \| |
| \|  \|  \| q604n_reasons (required) \| \| --- \| --- \| --- \| | Newborn resuscitation  Question relevant when: ${q604n} = '0' | \|  \| 1 \| No cases \| \| --- \| --- \| --- \| \|  \| 2 \| no supply \| \|  \| 3 \| Afraid of doing it \| \|  \| 4 \| Other \| |
| \|  \|  \| q604o_reasons (required) \| \| --- \| --- \| --- \| | Partograph use  Question relevant when: ${q604o} = '0' | \|  \| 1 \| No cases \| \| --- \| --- \| --- \| \|  \| 2 \| no supply \| \|  \| 3 \| Afraid of doing it \| \|  \| 4 \| Other \| |
| \|  \| q605 (required) \| \| --- \| --- \| | What are the primary aspects of focused antenatal care? Record all spontaneous answers  Prove: What else? | \|  \| 1 \| Minimum of 4 consultations \| \| --- \| --- \| --- \| \|  \| 2 \| Ensure woman has birth plan \| \|  \| 3 \| Prevent illness and promote health \| \|  \| 4 \| Detect existing illnesses and manage complications \| \|  \| 5 \| Teach the danger signs \| \|  \| 6 \| Promote breastfeeding \| |
| \|  \| q606 (required) \| \| --- \| --- \| | Which women require a special care plan? Record all spontaneous answers  Probe: What else? | \|  \| 1 \| Women who have had a cesarean \| \| --- \| --- \| --- \| \|  \| 2 \| Women with 5 or more deliveries \| \|  \| 3 \| Previous stillbirth \| \|  \| 4 \| Previous neonatal death \| \|  \| 5 \| Previous instrumental delivery \| \|  \| 6 \| History of severe obstetric complications \| \|  \| 7 \| Previous obstetric fistula repair \| |
| \|  \| q607 (required) \| \| --- \| --- \| | How do you know when a pregnant woman is in labor? Record all spontaneous answers  Probe: What else? | \|  \| 1 \| Regular uterine contractions \| \| --- \| --- \| --- \| \|  \| 2 \| Dilation of the cervix \| \|  \| 3 \| Discharge of blood and mucus \| \|  \| 4 \| Ruptured membranes \| |
| \|  \| q608 (required) \| \| --- \| --- \| | For a woman in labor, what observations do you make as you monitor her progress? Record all spontaneous answers  Probe: What else? | \|  \| 1 \| Fetal heartbeat \| \| --- \| --- \| --- \| \|  \| 2 \| Color of amniotic fluid \| \|  \| 3 \| Degree of molding \| \|  \| 4 \| Dilatation of the cervix \| \|  \| 5 \| Descent of the head \| \|  \| 6 \| Uterine contractions \| \|  \| 7 \| Maternal blood pressure \| \|  \| 8 \| Maternal temperature \| \|  \| 9 \| Maternal pulse \| \|  \| 10 \| Urine output, ketone and protein status \| |
| \|  \| q609 (required) \| \| --- \| --- \| | Where do you register these observations? record all spontaneous answers  Probe: What else? | \|  \| 1 \| On a partograph \| \| --- \| --- \| --- \| \|  \| 2 \| In the patient’s clinical record \| \|  \| 3 \| On a piece of paper \| |
| \|  \| q610 (required) \| \| --- \| --- \| | What are the actions taken during active management of the third stage of labor? Record all spontaneous answers  Probe: What else? | \|  \| 1 \| Immediate oxytocin/ergometrine (within 1 minutes) \| \| --- \| --- \| --- \| \|  \| 2 \| Controlled cord traction \| \|  \| 3 \| Uterine massage \| |
| \|  \| q611 (required) \| \| --- \| --- \| | The last time you delivered a baby, what immediate care did you give the newborn? Record all spontaneous answers  Probe: What else? | \|  \| 1 \| Clean the baby’s mouth before the shoulder comes out \| \| --- \| --- \| --- \| \|  \| 2 \| Clean the baby’s mouth, face, and nose \| \|  \| 3 \| Ensure the baby is breathing \| \|  \| 4 \| Ensure the baby is dry \| \|  \| 5 \| Observe for color \| \|  \| 6 \| Ensure baby is kept warm (skin-to-skin) \| \|  \| 7 \| Administer prophylaxis for the eyes \| \|  \| 8 \| Weigh the baby \| \|  \| 9 \| Care for the umbilical cord \| \|  \| 10 \| Initiate breastfeeding \| \|  \| 11 \| Provide Vitamin K \| \|  \| 12 \| Provide immunization \| \|  \| 13 \| Evaluate the newborn \| |
| \|  \| q612 (required) \| \| --- \| --- \| | When a woman arrives at the facility with heavy bleeding or develops severe bleeding after giving birth, what signs do you look for? Record all spontaneous answers  Probe: What else? | \|  \| 1 \| Signs of shock (dizziness, low blood pressure) \| \| --- \| --- \| --- \| \|  \| 2 \| Amount of external blood \| \|  \| 3 \| Signs of anemia \| \|  \| 4 \| Damage to the genital tract \| \|  \| 5 \| Whether the uterus is contracted \| \|  \| 6 \| Retained products or retained placenta \| \|  \| 7 \| Full bladder \| |
| \|  \| q613 (required) \| \| --- \| --- \| | When a woman develops heavy bleeding after delivery, what do you do? Record all spontaneous answers  Probe: What else? | \|  \| 1 \| Rapid evaluation \| \| --- \| --- \| --- \| \|  \| 2 \| Massage the fundus \| \|  \| 3 \| Give ergometrine or oxytocin (IV or IM) \| \|  \| 4 \| Begin IV fluids \| \|  \| 5 \| Empty full bladder \| \|  \| 6 \| Take blood for hgb and cross-matching \| \|  \| 7 \| Examine woman for lacerations \| \|  \| 8 \| Manually remove retained products \| \|  \| 9 \| Bimanual compression of aorta \| \|  \| 10 \| Refer \| |
| \|  \| q614 (required) \| \| --- \| --- \| | When a woman who just gave birth has a retained placenta, what do you do? Record all spontaneous answers  Probe: What else? | \|  \| 1 \| Empty the bladder \| \| --- \| --- \| --- \| \|  \| 2 \| CCT \| \|  \| 3 \| Give or repeat oxytocin \| \|  \| 4 \| Do manual removal of the placenta \| \|  \| 5 \| Administer IV fluids \| \|  \| 6 \| Monitor vital signs for shock and act \| \|  \| 7 \| Check that uterus is well contracted \| \|  \| 8 \| Determine blood type and cross-match \| \|  \| 9 \| Refer \| |
| \|  \| q615 (required) \| \| --- \| --- \| | What are the signs and symptoms of infection, or sepsis, in the newborn? Record all spontaneous answers  Probe: What else? | \|  \| 1 \| Less movement (poor muscle tone) \| \| --- \| --- \| --- \| \|  \| 2 \| Poor or no breastfeeding \| \|  \| 3 \| Hypothermia or hyperthermia \| \|  \| 4 \| Restlessness or irritability \| \|  \| 5 \| Difficulty breathing or fast breathing \| \|  \| 6 \| Deep jaundice \| \|  \| 7 \| Severe abdominal distention \| |
| \|  \| q616 (required) \| \| --- \| --- \| | When the newborn presents signs of infection, what initial steps do you take? Record all spontaneous answers  Probe: What else? | \|  \| 1 \| Explain the situation to the mother \| \| --- \| --- \| --- \| \|  \| 2 \| Continue to breastfeed or give breast milk that has been expressed with NG tube if necessary \| \|  \| 3 \| Keep airways open \| \|  \| 4 \| Begin antibiotics \| \|  \| 5 \| Refer \| |
| \|  \| q617 (required) \| \| --- \| --- \| | When a newborn weighs less than 2.5kgs, what special care do you provide? Record all spontaneous answers  Probe: What else? | \|  \| 1 \| Make sure the baby is warm/KMC \| \| --- \| --- \| --- \| \|  \| 2 \| Provide extra support to the mother to establish breastfeeding \| \|  \| 3 \| Monitor ability to breastfeed \| \|  \| 4 \| Monitor baby for the first 24 hours \| \|  \| 5 \| Ensure infection prevention \| |
| \|  \| q618 (required) \| \| --- \| --- \| | What are the immediate complications of an unsafe abortion? Record all spontaneous answers  Probe: What else? | \|  \| 1 \| Sepsis \| \| --- \| --- \| --- \| \|  \| 2 \| Bleeding \| \|  \| 3 \| Genital injuries \| \|  \| 4 \| Abdominal injuries \| \|  \| 5 \| Shock \| |
| \|  \| q619 (required) \| \| --- \| --- \| | When you see a woman with complications from an unsafe or incomplete abortion, what do you do? Record all spontaneous answers  Probe: What else? | \|  \| 1 \| Do a vaginal exam \| \| --- \| --- \| --- \| \|  \| 2 \| Assess vaginal bleeding \| \|  \| 3 \| Assess vital signs \| \|  \| 4 \| Begin IV fluids \| \|  \| 5 \| Begin antibiotics \| \|  \| 6 \| Do MVA or D&C or E&C \| \|  \| 7 \| Provide counseling \| \|  \| 8 \| Refer \| |
| \|  \| q620 (required) \| \| --- \| --- \| | What information do you give patients who were treated for an incomplete or unsafe abortion? Record all spontaneous answers  Probe: What else? | \|  \| 1 \| Information on how to prevent reproductive tract infection/HIV \| \| --- \| --- \| --- \| \|  \| 2 \| Information about when a woman can conceive again \| \|  \| 3 \| Counseling on family planning and services \| \|  \| 4 \| Provide FP methods \| \|  \| 5 \| Information on social support \| \|  \| 6 \| Information about the consequences of an unsafe abortion \| |
| \|  \| q621 (required) \| \| --- \| --- \| | Please describe how you would diagnose birth asphyxia. Record all spontaneous answers  Probe: What else? | \|  \| 1 \| Depressed breathing \| \| --- \| --- \| --- \| \|  \| 2 \| Floppiness \| \|  \| 3 \| Heart rate below 100 beats per minute \| \|  \| 4 \| Central cyanosis (blue tongue) \| |
| \|  \| q622 (required) \| \| --- \| --- \| | Please describe the sequential steps of neonatal resuscitation. Record all spontaneous answers  Probe: What else? | \|  \| 1 \| Call for help \| \| --- \| --- \| --- \| \|  \| 2 \| Wrap or cover baby, except for face and upper portion of chest \| \|  \| 3 \| Explain to mother condition of baby \| \|  \| 4 \| Position baby’s head so neck is slightly extended \| \|  \| 5 \| Suction mouth then nose \| \|  \| 6 \| Start ventilation using bag and mask \| |
| \|  \| q623 (required) \| \| --- \| --- \| | Were the steps mentioned in sequential order?  Do not ask; record observation | \|  \| 1 \| Yes \| \| --- \| --- \| --- \| \|  \| 0 \| No \| |
| \|  \| q624 (required) \| \| --- \| --- \| | If resuscitating with bag and mask or tube and mask, what do you do? Record all spontaneous answers  Probe: What else? | \|  \| 1 \| Place mask so it covers baby’s chin, mouth, and nose \| \| --- \| --- \| --- \| \|  \| 2 \| Ensure appropriate seal has been formed between mask, nose, mouth, and chin \| \|  \| 3 \| Ventilate 1 or 2 times and see if chest is rising \| \|  \| 4 \| Ventilate 40 times per minute for 1 minute \| \|  \| 5 \| Pause and determine whether baby is breathing spontaneously \| |
| \|  \| q625 (required) \| \| --- \| --- \| | If baby is breathing and there is no sign of respiratory difficulty (intercostal retractions or grunting), what do you do? Record all spontaneous answers  Probe: What else? | \|  \| 1 \| Keep baby warm \| \| --- \| --- \| --- \| \|  \| 2 \| Initiate breastfeeding \| \|  \| 3 \| Continue monitoring the baby \| |
| \|  \| q626 (required) \| \| --- \| --- \| | If baby does NOT begin to breathe or if breathing is less than 30 breaths per minute, or if there is intercostal retraction or grunting, what do you do? Record all spontaneous answers  Probe: What else? | \|  \| 1 \| Continue to ventilate \| \| --- \| --- \| --- \| \|  \| 2 \| Administer oxygen, if available \| \|  \| 3 \| Assess the need for special care \| \|  \| 4 \| Explain to the mother what is happening \| \|  \| 5 \| Intubate per adrenal resuscitation guidelines \| \|  \| 6 \| Refer the newborn \| |
| part3note | Part III: Partograph Review (Health center) |  |
| intronote3 | Instructions: Ask to see the partographs in use at the facility, and then ask the person in charge of the maternity the questions below. Make sure you are given copies of completed partographs to verify that the partograph is actually used in the facility. |  |
| q701 (required) | Do you use a partograph in this facility? | \|  \| 1 \| Yes \| \| --- \| --- \| --- \| \|  \| 0 \| No \| |
| Why do you not use the partograph in this facility?  Group relevant when: ${q701} = '0' | | |
| \|  \| q702a (required) \| \| --- \| --- \| | Staff are not trained | \|  \| 1 \| Yes \| \| --- \| --- \| --- \| \|  \| 0 \| No \| |
| \|  \| q702b (required) \| \| --- \| --- \| | No supplies (no blank partographs) | \|  \| 1 \| Yes \| \| --- \| --- \| --- \| \|  \| 0 \| No \| |
| \|  \| q702c (required) \| \| --- \| --- \| | Staff do not have time | \|  \| 1 \| Yes \| \| --- \| --- \| --- \| \|  \| 0 \| No \| |
| \|  \| q702d (required) \| \| --- \| --- \| | Use of the partograph is not mandatory/not policy | \|  \| 1 \| Yes \| \| --- \| --- \| --- \| \|  \| 0 \| No \| |
| \|  \| q702e (required) \| \| --- \| --- \| | Other | \|  \| 1 \| Yes \| \| --- \| --- \| --- \| \|  \| 0 \| No \| |
| partographreview  Group relevant when: ${q701} = '1' | | |
| \|  \| q703 (required) \| \| --- \| --- \| | Is there a labor management protocol for a woman using a partograph? (ask and then confirm by observation of poster/chart on the wall) | \|  \| 1 \| Yes \| \| --- \| --- \| --- \| \|  \| 0 \| No \| |
| \|  \| partogragh_review \| \| --- \| --- \| | Now; Look through recent records and select three recent partographs filled out by different providers, if possible. Also ask for the case notes and/or patient records for these partographs. The partographs should belong to women with the following characteristics: at term, <8cm dilatation at first exam, vertex presentation, fetal heart present at first exam, and without obstetric complications at first exam. Select only partographs that start with the active phase of labor. If the answer to Item 705 is “Not assessable,” do not select another partograph to replace this one. There is one column for each partograph. Complete the codes/answers for each question, answering all the questions for one partograph, and then go to the second partograph, and the third partograph. |  |
| \|  \| introcase1 \| \| --- \| --- \| | Case I  Record review of case 1 |  |
| \|  \| q7041 (required) \| \| --- \| --- \| | What was the woman’s dilatation when the partograph was started?  Write number of centimeters  Response constrained to: .>=4 and .<=8 |  |
| \|  \| q7051 (required) \| \| --- \| --- \| | Was the first dilatation charted on the alert line?  If “No,” end the review for this case; partograph is not assessable | \|  \| 1 \| Yes \| \| --- \| --- \| --- \| \|  \| 0 \| No \| |
| \|  \| partographreview > Partograph assessable  Group relevant when: ${q7051} = '1' \| \| --- \| --- \| | | |
| \|  \|  \| partographreview > Partograph assessable > How many hours and minutes elapsed between first exam and delivery? \| \| --- \| --- \| --- \| | | |
| \|  \|  \|  \| q7061a \| \| --- \| --- \| --- \| --- \| | Hours? |  |
| \|  \|  \|  \| q7061b \| \| --- \| --- \| --- \| --- \| | Minutes? |  |
| \|  \|  \| q7071 (required) \| \| --- \| --- \| --- \| | How many times was the woman’s temperature checked and recorded between first exam and delivery (including first exam and delivery)? |  |
| \|  \|  \| q7081 (required) \| \| --- \| --- \| --- \| | How many times was blood pressure checked and recorded between first exam and delivery (including first exam and delivery)? |  |
| \|  \|  \| q7091 (required) \| \| --- \| --- \| --- \| | How many times was the mother’s pulse checked and recorded between first exam and delivery (including first exam and delivery)? |  |
| \|  \|  \| q7101 (required) \| \| --- \| --- \| --- \| | Was the fetal heart rate observed at least at hourly intervals? | \|  \| 1 \| Yes \| \| --- \| --- \| --- \| \|  \| 0 \| No \| |
| \|  \|  \| q7111 (required) \| \| --- \| --- \| --- \| | Were contractions assessed at least hourly? | \|  \| 1 \| Yes \| \| --- \| --- \| --- \| \|  \| 0 \| No \| |
| \|  \|  \| q7121 (required) \| \| --- \| --- \| --- \| | How many times was a vaginal examination carried out and recorded between first exam and delivery (including first exam and delivery)? |  |
| \|  \|  \| q7131 (required) \| \| --- \| --- \| --- \| | Was the descent checked and recorded between first exam and delivery? | \|  \| 1 \| Yes \| \| --- \| --- \| --- \| \|  \| 0 \| No \| |
| \|  \|  \| q7141 (required) \| \| --- \| --- \| --- \| | Was the state of the membranes or color of the liquor recorded? | \|  \| 1 \| Yes \| \| --- \| --- \| --- \| \|  \| 0 \| No \| |
| \|  \|  \| q7151 (required) \| \| --- \| --- \| --- \| | According to the partograph, when did the woman deliver? | \|  \| 1 \| On or left of the alert line \| \| --- \| --- \| --- \| \|  \| 2 \| Between the alert and action lines \| \|  \| 3 \| On or to the right of the action line \| |
| \|  \|  \| partographreview > Partograph assessable > If she delivered on or to the right of the action line, how many hours and minutes to the right of the action line?  Group relevant when: ${q7151} = '1' or ${q7151} = '2' \| \| --- \| --- \| --- \| | | |
| \|  \|  \|  \| q7161a \| \| --- \| --- \| --- \| --- \| | Hours? |  |
| \|  \|  \|  \| q7161b \| \| --- \| --- \| --- \| --- \| | Minutes? |  |
| \|  \|  \| q7171 (required) \| \| --- \| --- \| --- \| | Was augmentation used? | \|  \| 1 \| Yes \| \| --- \| --- \| --- \| \|  \| 0 \| No \| |
| \|  \|  \| q7181 (required) \| \| --- \| --- \| --- \| | If augmentation was used, when?  Question relevant when: ${q7171} = '1' | \|  \| 1 \| On the alert line \| \| --- \| --- \| --- \| \|  \| 2 \| Between the alert and action lines \| \|  \| 3 \| On or beyond the action line \| |
| \|  \|  \| q7191 (required) \| \| --- \| --- \| --- \| | Was time at delivery filled in?  Refer to the case notes/patient record if necessary | \|  \| 1 \| Yes \| \| --- \| --- \| --- \| \|  \| 0 \| No \| |
| \|  \|  \| q7201 (required) \| \| --- \| --- \| --- \| | What type of delivery did she have?  Refer to the case notes/patient record if necessary | \|  \| 1 \| Spontaneous vertex delivery \| \| --- \| --- \| --- \| \|  \| 2 \| Vacuum extraction \| \|  \| 88 \| Other \| \|  \| 99 \| No information \| |
| \|  \|  \| q7201_oth (required) \| \| --- \| --- \| --- \| | Please specify other  Question relevant when: ${q7201} = '1' |  |
| \|  \|  \| q7211 (required) \| \| --- \| --- \| --- \| | If delivery by vacuum, state reason.  Refer to the case notes/patient record if necessary  Question relevant when: ${q7201} = '2' | \|  \| 1 \| Cephalopelvic disproportion (CPD) \| \| --- \| --- \| --- \| \|  \| 2 \| Fetal distress \| \|  \| 88 \| Other \| |
| \|  \|  \| q7211_oth (required) \| \| --- \| --- \| --- \| | Please specify other |  |
| \|  \|  \| q7221 (required) \| \| --- \| --- \| --- \| | What was the outcome for the baby?  Refer to the case notes/patient record if necessary | \|  \| 1 \| Normal live birth \| \| --- \| --- \| --- \| \|  \| 2 \| Live birth with distress \| \|  \| 3 \| Stillbirth \| \|  \| 99 \| No information \| |
| \|  \| introcase2 \| \| --- \| --- \| | Case 2  Record review of case 2 |  |
| \|  \| q7042 (required) \| \| --- \| --- \| | What was the woman’s dilatation when the partograph was started?  Write number of centimeters  Response constrained to: .>=4 and .<=8 |  |
| \|  \| q7052 (required) \| \| --- \| --- \| | Was the first dilatation charted on the alert line?  If “No,” end the review for this case; partograph is not assessable | \|  \| 1 \| Yes \| \| --- \| --- \| --- \| \|  \| 0 \| No \| |
| \|  \| partographreview > Partograph assessable  Group relevant when: ${q7052} = '1' \| \| --- \| --- \| | | |
| \|  \|  \| partographreview > Partograph assessable > How many hours and minutes elapsed between first exam and delivery? \| \| --- \| --- \| --- \| | | |
| \|  \|  \|  \| q7062a \| \| --- \| --- \| --- \| --- \| | Hours? |  |
| \|  \|  \|  \| q7062b \| \| --- \| --- \| --- \| --- \| | Minutes? |  |
| \|  \|  \| q7072 (required) \| \| --- \| --- \| --- \| | How many times was the woman’s temperature checked and recorded between first exam and delivery (including first exam and delivery)? |  |
| \|  \|  \| q7082 (required) \| \| --- \| --- \| --- \| | How many times was blood pressure checked and recorded between first exam and delivery (including first exam and delivery)? |  |
| \|  \|  \| q7092 (required) \| \| --- \| --- \| --- \| | How many times was the mother’s pulse checked and recorded between first exam and delivery (including first exam and delivery)? |  |
| \|  \|  \| q7102 (required) \| \| --- \| --- \| --- \| | Was the fetal heart rate observed at least at hourly intervals? | \|  \| 1 \| Yes \| \| --- \| --- \| --- \| \|  \| 0 \| No \| |
| \|  \|  \| q7112 (required) \| \| --- \| --- \| --- \| | Were contractions assessed at least hourly? | \|  \| 1 \| Yes \| \| --- \| --- \| --- \| \|  \| 0 \| No \| |
| \|  \|  \| q7122 (required) \| \| --- \| --- \| --- \| | How many times was a vaginal examination carried out and recorded between first exam and delivery (including first exam and delivery)? |  |
| \|  \|  \| q7132 (required) \| \| --- \| --- \| --- \| | Was the descent checked and recorded between first exam and delivery? | \|  \| 1 \| Yes \| \| --- \| --- \| --- \| \|  \| 0 \| No \| |
| \|  \|  \| q7142 (required) \| \| --- \| --- \| --- \| | Was the state of the membranes or color of the liquor recorded? | \|  \| 1 \| Yes \| \| --- \| --- \| --- \| \|  \| 0 \| No \| |
| \|  \|  \| q7152 (required) \| \| --- \| --- \| --- \| | According to the partograph, when did the woman deliver? | \|  \| 1 \| On or left of the alert line \| \| --- \| --- \| --- \| \|  \| 2 \| Between the alert and action lines \| \|  \| 3 \| On or to the right of the action line \| |
| \|  \|  \| partographreview > Partograph assessable > If she delivered on or to the right of the action line, how many hours and minutes to the right of the action line?  Group relevant when: ${q7152} = '1' or ${q7152} = '2' \| \| --- \| --- \| --- \| | | |
| \|  \|  \|  \| q7162a \| \| --- \| --- \| --- \| --- \| | Hours? |  |
| \|  \|  \|  \| q7162b \| \| --- \| --- \| --- \| --- \| | Minutes? |  |
| \|  \|  \| q7172 (required) \| \| --- \| --- \| --- \| | Was augmentation used? | \|  \| 1 \| Yes \| \| --- \| --- \| --- \| \|  \| 0 \| No \| |
| \|  \|  \| q7182 (required) \| \| --- \| --- \| --- \| | If augmentation was used, when?  Question relevant when: ${q7172} = '1' | \|  \| 1 \| On the alert line \| \| --- \| --- \| --- \| \|  \| 2 \| Between the alert and action lines \| \|  \| 3 \| On or beyond the action line \| |
| \|  \|  \| q7192 (required) \| \| --- \| --- \| --- \| | Was time at delivery filled in?  Refer to the case notes/patient record if necessary | \|  \| 1 \| Yes \| \| --- \| --- \| --- \| \|  \| 0 \| No \| |
| \|  \|  \| q7202 (required) \| \| --- \| --- \| --- \| | What type of delivery did she have?  Refer to the case notes/patient record if necessary | \|  \| 1 \| Spontaneous vertex delivery \| \| --- \| --- \| --- \| \|  \| 2 \| Vacuum extraction \| \|  \| 88 \| Other \| \|  \| 99 \| No information \| |
| \|  \|  \| q7202_oth (required) \| \| --- \| --- \| --- \| | Please specify other  Question relevant when: ${q7202} = '1' |  |
| \|  \|  \| q7212 (required) \| \| --- \| --- \| --- \| | If delivery by vacuum, state reason.  Refer to the case notes/patient record if necessary  Question relevant when: ${q7202} = '2' | \|  \| 1 \| Cephalopelvic disproportion (CPD) \| \| --- \| --- \| --- \| \|  \| 2 \| Fetal distress \| \|  \| 88 \| Other \| |
| \|  \|  \| q7212_oth (required) \| \| --- \| --- \| --- \| | Please specify other |  |
| \|  \|  \| q7222 (required) \| \| --- \| --- \| --- \| | What was the outcome for the baby?  Refer to the case notes/patient record if necessary | \|  \| 1 \| Normal live birth \| \| --- \| --- \| --- \| \|  \| 2 \| Live birth with distress \| \|  \| 3 \| Stillbirth \| \|  \| 99 \| No information \| |
| \|  \| introcase3 \| \| --- \| --- \| | Case 3  Record review of case 3 |  |
| \|  \| q7043 (required) \| \| --- \| --- \| | What was the woman’s dilatation when the partograph was started?  Write number of centimeters  Response constrained to: .>=4 and .<=8 |  |
| \|  \| q7053 (required) \| \| --- \| --- \| | Was the first dilatation charted on the alert line?  If “No,” end the review for this case; partograph is not assessable | \|  \| 1 \| Yes \| \| --- \| --- \| --- \| \|  \| 0 \| No \| |
| \|  \| partographreview > Partograph assessable  Group relevant when: ${q7053} = '1' \| \| --- \| --- \| | | |
| \|  \|  \| partographreview > Partograph assessable > How many hours and minutes elapsed between first exam and delivery? \| \| --- \| --- \| --- \| | | |
| \|  \|  \|  \| q7063a \| \| --- \| --- \| --- \| --- \| | Hours? |  |
| \|  \|  \|  \| q7063b \| \| --- \| --- \| --- \| --- \| | Minutes? |  |
| \|  \|  \| q7073 (required) \| \| --- \| --- \| --- \| | How many times was the woman’s temperature checked and recorded between first exam and delivery (including first exam and delivery)? |  |
| \|  \|  \| q7083 (required) \| \| --- \| --- \| --- \| | How many times was blood pressure checked and recorded between first exam and delivery (including first exam and delivery)? |  |
| \|  \|  \| q7093 (required) \| \| --- \| --- \| --- \| | How many times was the mother’s pulse checked and recorded between first exam and delivery (including first exam and delivery)? |  |
| \|  \|  \| q7103 (required) \| \| --- \| --- \| --- \| | Was the fetal heart rate observed at least at hourly intervals? | \|  \| 1 \| Yes \| \| --- \| --- \| --- \| \|  \| 0 \| No \| |
| \|  \|  \| q7113 (required) \| \| --- \| --- \| --- \| | Were contractions assessed at least hourly? | \|  \| 1 \| Yes \| \| --- \| --- \| --- \| \|  \| 0 \| No \| |
| \|  \|  \| q7123 (required) \| \| --- \| --- \| --- \| | How many times was a vaginal examination carried out and recorded between first exam and delivery (including first exam and delivery)? |  |
| \|  \|  \| q7133 (required) \| \| --- \| --- \| --- \| | Was the descent checked and recorded between first exam and delivery? | \|  \| 1 \| Yes \| \| --- \| --- \| --- \| \|  \| 0 \| No \| |
| \|  \|  \| q7143 (required) \| \| --- \| --- \| --- \| | Was the state of the membranes or color of the liquor recorded? | \|  \| 1 \| Yes \| \| --- \| --- \| --- \| \|  \| 0 \| No \| |
| \|  \|  \| q7153 (required) \| \| --- \| --- \| --- \| | According to the partograph, when did the woman deliver? | \|  \| 1 \| On or left of the alert line \| \| --- \| --- \| --- \| \|  \| 2 \| Between the alert and action lines \| \|  \| 3 \| On or to the right of the action line \| |
| \|  \|  \| partographreview > Partograph assessable > If she delivered on or to the right of the action line, how many hours and minutes to the right of the action line?  Group relevant when: ${q7153} = '1' or ${q7153} = '2' \| \| --- \| --- \| --- \| | | |
| \|  \|  \|  \| q7163a (required) \| \| --- \| --- \| --- \| --- \| | Hours? |  |
| \|  \|  \|  \| q7163b (required) \| \| --- \| --- \| --- \| --- \| | Minutes? |  |
| \|  \|  \| q7173 (required) \| \| --- \| --- \| --- \| | Was augmentation used? | \|  \| 1 \| Yes \| \| --- \| --- \| --- \| \|  \| 0 \| No \| |
| \|  \|  \| q7183 (required) \| \| --- \| --- \| --- \| | If augmentation was used, when?  Question relevant when: ${q7173} = '1' | \|  \| 1 \| On the alert line \| \| --- \| --- \| --- \| \|  \| 2 \| Between the alert and action lines \| \|  \| 3 \| On or beyond the action line \| |
| \|  \|  \| q7193 (required) \| \| --- \| --- \| --- \| | Was time at delivery filled in?  Refer to the case notes/patient record if necessary | \|  \| 1 \| Yes \| \| --- \| --- \| --- \| \|  \| 0 \| No \| |
| \|  \|  \| q7203 (required) \| \| --- \| --- \| --- \| | What type of delivery did she have?  Refer to the case notes/patient record if necessary | \|  \| 1 \| Spontaneous vertex delivery \| \| --- \| --- \| --- \| \|  \| 2 \| Vacuum extraction \| \|  \| 88 \| Other \| \|  \| 99 \| No information \| |
| \|  \|  \| q7203_oth (required) \| \| --- \| --- \| --- \| | Please specify other  Question relevant when: ${q7203} = '1' |  |
| \|  \|  \| q7213 (required) \| \| --- \| --- \| --- \| | If delivery by vacuum, state reason.  Refer to the case notes/patient record if necessary  Question relevant when: ${q7203} = '2' | \|  \| 1 \| Cephalopelvic disproportion (CPD) \| \| --- \| --- \| --- \| \|  \| 2 \| Fetal distress \| \|  \| 88 \| Other \| |
| \|  \|  \| q7213_oth (required) \| \| --- \| --- \| --- \| | Please specify other |  |
| \|  \|  \| q7223 (required) \| \| --- \| --- \| --- \| | What was the outcome for the baby?  Refer to the case notes/patient record if necessary | \|  \| 1 \| Normal live birth \| \| --- \| --- \| --- \| \|  \| 2 \| Live birth with distress \| \|  \| 3 \| Stillbirth \| \|  \| 99 \| No information \| |
| Service statistics (1) | | (Repeated group) |
| \|  \| month (required) \| \| --- \| --- \| | Reporting month | \|  \| 1 \| Hamle 2006 \| \| --- \| --- \| --- \| \|  \| 2 \| Nehase 2006 \| \|  \| 3 \| Meskerem 2007 \| \|  \| 4 \| Tikmet 2007 \| \|  \| 5 \| Hidar 2007 \| \|  \| 6 \| Tahsas 2007 \| \|  \| 7 \| Tir 2007 \| \|  \| 8 \| Yekatit 2007 \| \|  \| 9 \| Megabit 2007 \| \|  \| 10 \| Miazia 2007 \| \|  \| 11 \| Ginbot 2007 \| \|  \| 12 \| Sene 2007 \| |
| \|  \| q805 (required) \| \| --- \| --- \| | Total Number of deliveries attended  Record "999" if no data |  |
| \|  \| q805a (required) \| \| --- \| --- \| | Assisted deliveries  Record "999" if no data  Response constrained to: (. = ${q805} ) or (. < ${q805} ) |  |
| \|  \| q806 (required) \| \| --- \| --- \| | Number of Live births  Record "999" if no data  Response constrained to: (. = ${q805} ) or (. > ${q805} ) |  |
| \|  \| q807 (required) \| \| --- \| --- \| | Number of Stillbirths  Record "999" if no data  Response constrained to: (. = ${q805} ) or (. < ${q805} ) |  |
| \|  \| q808 (required) \| \| --- \| --- \| | Number of early neonatal deaths  Record "999" if no data  Response constrained to: (. = ${q805} ) or (. < ${q805} ) |  |
| \|  \| q809 (required) \| \| --- \| --- \| | No. of neonatal deaths  Record "999" if no data  Response constrained to: (. = ${q805} ) or (. < ${q805} ) |  |
| \|  \| q810 (required) \| \| --- \| --- \| | No. of sick young infants managed  Record "999" if no data |  |
| \|  \| q811 (required) \| \| --- \| --- \| | No. of young infant deaths  Record "999" if no data |  |
| \|  \| q812 (required) \| \| --- \| --- \| | No. of maternal deaths  Record "999" if no data  Response constrained to: (. = ${q805} ) or (. < ${q805} ) |  |
| \|  \| Service statistics (1) > Obstetric complications managed at health center \| \| --- \| --- \| | | |
| \|  \|  \| q813a (required) \| \| --- \| --- \| --- \| | Abortion complications |  |
| \|  \|  \| q813b (required) \| \| --- \| --- \| --- \| | PPH |  |
| \|  \|  \| q813c (required) \| \| --- \| --- \| --- \| | Obstructed/ prolonged labor |  |
| \|  \|  \| q813d (required) \| \| --- \| --- \| --- \| | Retained placenta |  |
| \|  \|  \| q813e (required) \| \| --- \| --- \| --- \| | Puerperal sepsis |  |
| \|  \| Service statistics (1) > Adherence to protocols for maternal referrals \| \| --- \| --- \| | | |
| \|  \|  \| q801 (required) \| \| --- \| --- \| --- \| | Number of urgent maternal referral cases received (managed & referred) during the reporting month |  |
| \|  \|  \| q801a (required) \| \| --- \| --- \| --- \| | Self referral |  |
| \|  \|  \| q801b (required) \| \| --- \| --- \| --- \| | Arrived with referral slip |  |
| \|  \|  \| q801c (required) \| \| --- \| --- \| --- \| | Used Ambulance |  |
| \|  \|  \| q801d (required) \| \| --- \| --- \| --- \| | Called ahead |  |
| \|  \|  \| q801e (required) \| \| --- \| --- \| --- \| | Escorted (by HEWs, auxiliary staff or HW) |  |
| \|  \|  \| q801f (required) \| \| --- \| --- \| --- \| | Provided feedback |  |
| \|  \| Service statistics (1) > Adherence to protocols for newborn referrals \| \| --- \| --- \| | | |
| \|  \|  \| q802 (required) \| \| --- \| --- \| --- \| | Number of sick young infant (0 - 2 months) referral cases received (managed & referred) during the reporting month |  |
| \|  \|  \| q802a (required) \| \| --- \| --- \| --- \| | Self referral |  |
| \|  \|  \| q802b (required) \| \| --- \| --- \| --- \| | Arrived with referral slip |  |
| \|  \|  \| q802c (required) \| \| --- \| --- \| --- \| | Used Ambulance |  |
| \|  \|  \| q802d (required) \| \| --- \| --- \| --- \| | Called ahead |  |
| \|  \|  \| q802e (required) \| \| --- \| --- \| --- \| | Escorted (by HEWs, auxiliary staff or HW) |  |
| \|  \|  \| q802f (required) \| \| --- \| --- \| --- \| | Provided feedback |  |
| \|  \| q803 (required) \| \| --- \| --- \| | Median time interval between HP and health center (for maternal referral cases)  Record in minutes |  |
| \|  \| q804 (required) \| \| --- \| --- \| | Number urgent maternal referrals reached designated hospital  Record "999" if no data |  |
| \|  \| Service statistics (1) > Total number of obstetric emergencies who have been given a ride/transportation in the past one year? \| \| --- \| --- \| | | |
| \|  \|  \| q814a (required) \| \| --- \| --- \| --- \| | From community to HC |  |
| \|  \|  \| q814b (required) \| \| --- \| --- \| --- \| | From HC to hospital |  |
| \|  \| Service statistics (1) > Total number of neonatal emergencies who have been given a ride/transportation in the past one year? \| \| --- \| --- \| | | |
| \|  \|  \| q815a (required) \| \| --- \| --- \| --- \| | From community to HC |  |
| \|  \|  \| q815b (required) \| \| --- \| --- \| --- \| | From HC to hospital |  |
| \|  \| Service statistics (1) > Total number of non-obstetric emergencies who have been given a ride/transportation in the past one year? \| \| --- \| --- \| | | |
| \|  \|  \| q816a (required) \| \| --- \| --- \| --- \| | From community to HC |  |
| \|  \|  \| q816b (required) \| \| --- \| --- \| --- \| | From HC to hospital |  |
| \|  \| introworeda \| \| --- \| --- \| | Service utilization of the woreda in the last 12 months |  |
| \|  \| Service statistics (1) > Total number of obstetric emergencies who have been given a ride/transportation in the past one year in the woreda? \| \| --- \| --- \| | | |
| \|  \|  \| q817a (required) \| \| --- \| --- \| --- \| | From community to HC |  |
| \|  \|  \| q817b (required) \| \| --- \| --- \| --- \| | From HC to hospital |  |
| \|  \| Service statistics (1) > Total number of newborn emergencies who have been given a ride/transportation in the past one year in the woreda? \| \| --- \| --- \| | | |
| \|  \|  \| q818a (required) \| \| --- \| --- \| --- \| | From community to HC |  |
| \|  \|  \| q818b (required) \| \| --- \| --- \| --- \| | From HC to hospital |  |
| \|  \| Service statistics (1) > Total number of non-obstetric emergencies who have been given a ride/transportation in the past one year in the woreda? \| \| --- \| --- \| | | |
| \|  \|  \| q819a (required) \| \| --- \| --- \| --- \| | From community to HC |  |
| \|  \|  \| q819b (required) \| \| --- \| --- \| --- \| | From HC to hospital |  |
